# Homo-Oligomerisation in Signal Transduction: Dynamics, Homeostasis, Ultrasensitivity, Bistability

**DOI:** 10.1016/j.jtbi.2020.110305

**Published:** 2020-08-21

**Authors:** Daniel Koch

**Affiliations:** Randall Centre for Cell & Molecular Biophysics King’s College London, London SE1 1UL, United Kingdom

**Keywords:** Protein complexes, Mathematical modelling, Dynamic signal encoding, Post-translational modifications, Multi-enzyme systems

## Abstract

Homo-oligomerisation of proteins is a ubiquitous phenomenon whose exact role remains unclear in many cases. To identify novel functions, this paper provides an exploration of general dynamical mathematical models of homo-oligomerisation. Simulation and analysis of these models show that homo-oligomerisation on its own allows for a remarkable variety of complex dynamic and steady state regulatory behaviour such as transient overshoots or homeostatic control of monomer concentration. If post-translational modifications are considered, however, conventional mass action kinetics lead to thermodynamic inconsistencies due to asymmetric combinatorial expansion of reaction routes. Introducing a conservation principle to balance rate equations re-establishes thermodynamic consistency. Using such balanced models it is shown that oligomerisation can lead to bistability by enabling pseudo-multisite modification and kinetic pseudo-cooperativity via multi-enzyme regulation, thereby constituting a novel motif for bistable modification reactions. Due to these potential signal processing capabilities, homo-oligomerisation could play far more versatile roles in signal transduction than previously appreciated.

## Highlights

• Homo-oligomerisation may offer an unexpected variety of signal processing functions.
• These include: dynamic signal encoding via oligomeric complex size.
• Homeostatic regulation of monomer concentration.
• Bistability via pseudo-multisite modification and multi-enzyme regulation.
• The findings partly explain why homo-oligomerisation is so commonly found in evolution.

## Graphical abstract

**Figure:**
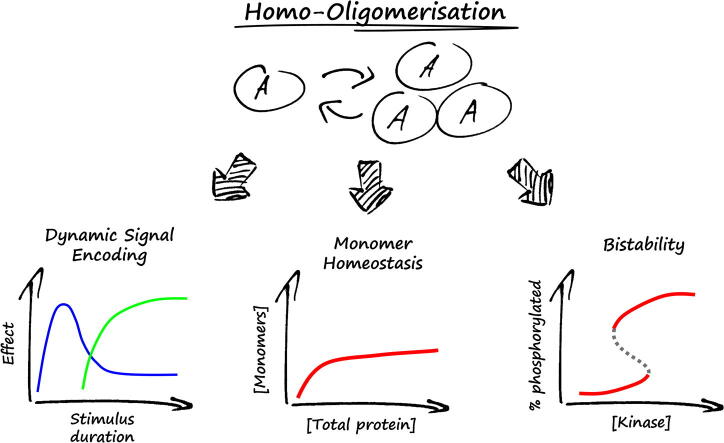

## 1 Introduction

Homo-oligomerisation of proteins, i.e. the assembly of supramolecular protein complexes made up from multiple identical subunits, is a ubiquitous phenomenon. In vertebrates, about 30–50% of all proteins form homo-oligomers, most of which are dimers (≈72%), tetramers (≈17%) and trimers (≈8%), while only ≈3% form other higher order oligomers (Lynch, 2012, Marsh and Teichmann, 2015). Oligomerisation may offer several advantages: it can be a way to economically assemble larger structures (thereby reducing genome size) and allows for a higher error-free transcription chance for individual subunits. Moreover, it can provide additional regulatory control via allostery and cooperative binding events (hemoglobin being the classical example) (Marianayagam et al., 2004, Ali and Imperiali, 2005). Yet, in many cases the function of homo-oligomerisation remains unclear.

Dynamical mathematical models based on ordinary differential equations (ODEs) have been extensively used to study many important motifs, mechanisms and phenomena in signal transduction networks. To lesser extent, ODE models have also been used to study signal transduction processes involving homo-oligomers. Such theoretical studies have shown that in addition to the well-known role in the emergence of ultrasensitive responses via cooperative binding, oligomerisation can provide an additional layer of control over such responses. Bouhaddou and Birtwistle, for instance, showed that different oligomerisation routes provide an effective means of tuning ultrasensitive, cooperative responses (Bouhaddou and Birtwistle, 2014). Buchler and Louis showed that homo-oligomerisation itself can lead to modest ultrasensitivity independent from cooperativity (Buchler and Louis, 2008). If coupled to positive feedback, the ultrasensitivity generated e.g. by homo-dimerisation is sufficient for the emergence of bistability (Hsu et al., 2016). For signalling involving dimeric receptors and substrate activation, the presence of a single/dual activation mechanism can lead to complex, non-linear signal dynamics (Vera et al., 2008). Taken together, this highlights the importance of homo-oligomerisation and the use of mathematical modelling as a tool to study its roles in signal transduction.

However, above mentioned studies focussed on specific questions, contexts or systems. A general analysis of homo-oligomerisation in terms of assembly dynamics, steady state behaviour and the potential effects of post-translational modifications (PTMs) is neither covered by classical and popular textbooks on mathematical or system’s biology (see e.g. Murray, 2002, Klipp et al., 2005, Keshet-Edelstein, 2005, Voit, 2012, IIngalls, 2013), nor is the author aware of such analysis in the recent research literature. It thus seems that an exploration of general dynamical mathematical models of homo-oligomerisation is still lacking. This paper provides such an exploration. As this study focusses solely on homo-oligomerisation, we will often leave out the prefix ‘homo-’ in the remainder of this article for the sake of brevity.

Beginning with simple mass action kinetics based models of dimerisation to tetramerisation, we will study assembly dynamics and steady state behaviour numerically. Although the first presented models are very simple, it is found that they are capable of complex dynamic and steady state behaviour such as undulations and homeostatic regulation.

Next, PTMs of oligomers are considered. Surprisingly, the application of conventional mass action rate laws easily results in thermodynamically inconsistent models due to combinatorial expansion of the oligomerisation routes upon modification. To keep the focus on biological results, details on this technical issue and how it can be circumvented are discussed in the supplement.

Finally, two novel mechanisms based on oligomerisation leading to ultrasensitive, bistable PTM responses will be presented: pseudo-multisite modification and regulation by multiple enzymes.

The focus of the current work is to demonstrate that oligomerisation enables more complex regulatory behaviour than previously appreciated. While the broad scope of a general analysis of dynamical mathematical models of oligomerisation does not permit an exhaustive treatment of all aspects within the limit of a single article, some of the most important implications and avenues for future research will be outlined in the discussion.

## 2 Results

### 2.1 Simple mass action models of oligomerisation: transients and homeostasis

Let us begin by assuming that a general protein *A* can form symmetric oligomers with a maximum number of *n* subunits (protomers) per oligomeric complex. We furthermore assume that *A* can form all intermediate oligomeric species with *m* subunits (where m∈N,1<m<n) and that each oligomeric species is formed through simple one-step, second-order binding reactions described by mass action kinetics. For the remainder of this article, we will study oligomers with a maximum of four protomers or less, i.e. tetramers, trimers and dimers. It is likely that many of the presented findings apply to higher-order oligomers as well.

**Fig. 1:**
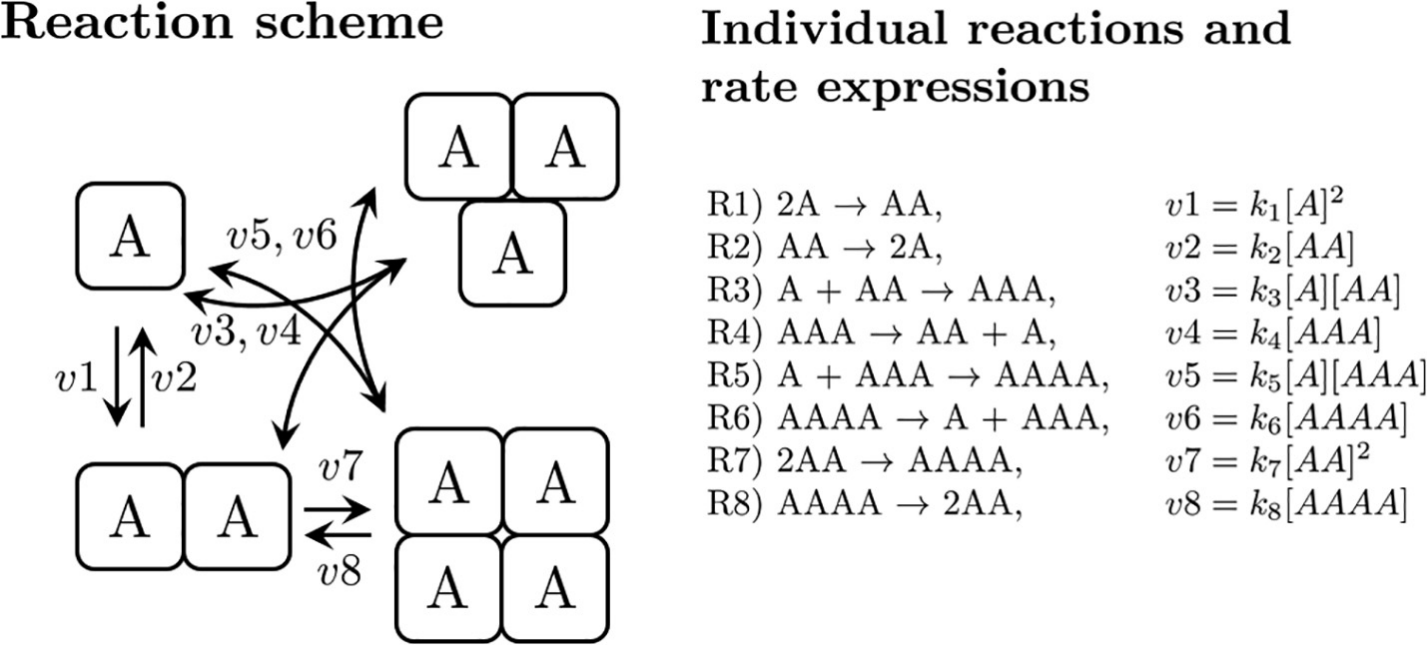
Model scheme of homo-tetramerisation based on conventional mass action kinetics assuming that all intermediate species (dimers and trimers) are possible in the reaction pathway. See text for the differential equations describing the system.

In the case of tetramers, we therefore assume that tetramers can be formed by the association of two dimers or, alternatively, of a trimer and a monomer. The reaction scheme and the reaction rates for the individual reactions can then be summarised as in Fig. 1. Denoting the monomeric to tetrameric species by A,…,AAAA we can now formulate the system’s equations:

$$\begin{array}{cc} & \frac{d}{\mathrm{dt}}[A]=2\cdot v2+v4+v6-2\cdot v1-v3-v5\\ & \frac{d}{\mathrm{dt}}[\mathrm{AA}]=v1+v4+2\cdot v8-v2-v3-2\cdot v7\\ & \frac{d}{\mathrm{dt}}[\mathrm{AAA}]=v3+v6-v4-v5\\ & \frac{d}{\mathrm{dt}}[\mathrm{AAAA}]=v5+v7-v6-v8 \end{array}$$

The total amount of subunits is conserved by the relation $A_{\mathrm{tot}}=[A]+2\cdot [\mathrm{AA}]+3\cdot [\mathrm{AAA}]+4\cdot [\mathrm{AAAA}]$ which can be used to eliminate one of the above equations. Note that models for tri- or dimerisation can be obtained simply by removing reactions R5-R8 or R3-R8, respectively. For the sake of simplicity, we will begin by assuming equal rate constants of $10^{6}\;{\text{mol}}^{-1}\;{\text{s}}^{-1}$ for all association reactions and equal rate constants of $0.1\;{\text{s}}^{-1}$ for all dissociation reactions, thereby yielding a $K_{d}$ value of 0.1 μM for all reactions, a typical value for many protein–protein interactions.

**Fig. 2:**
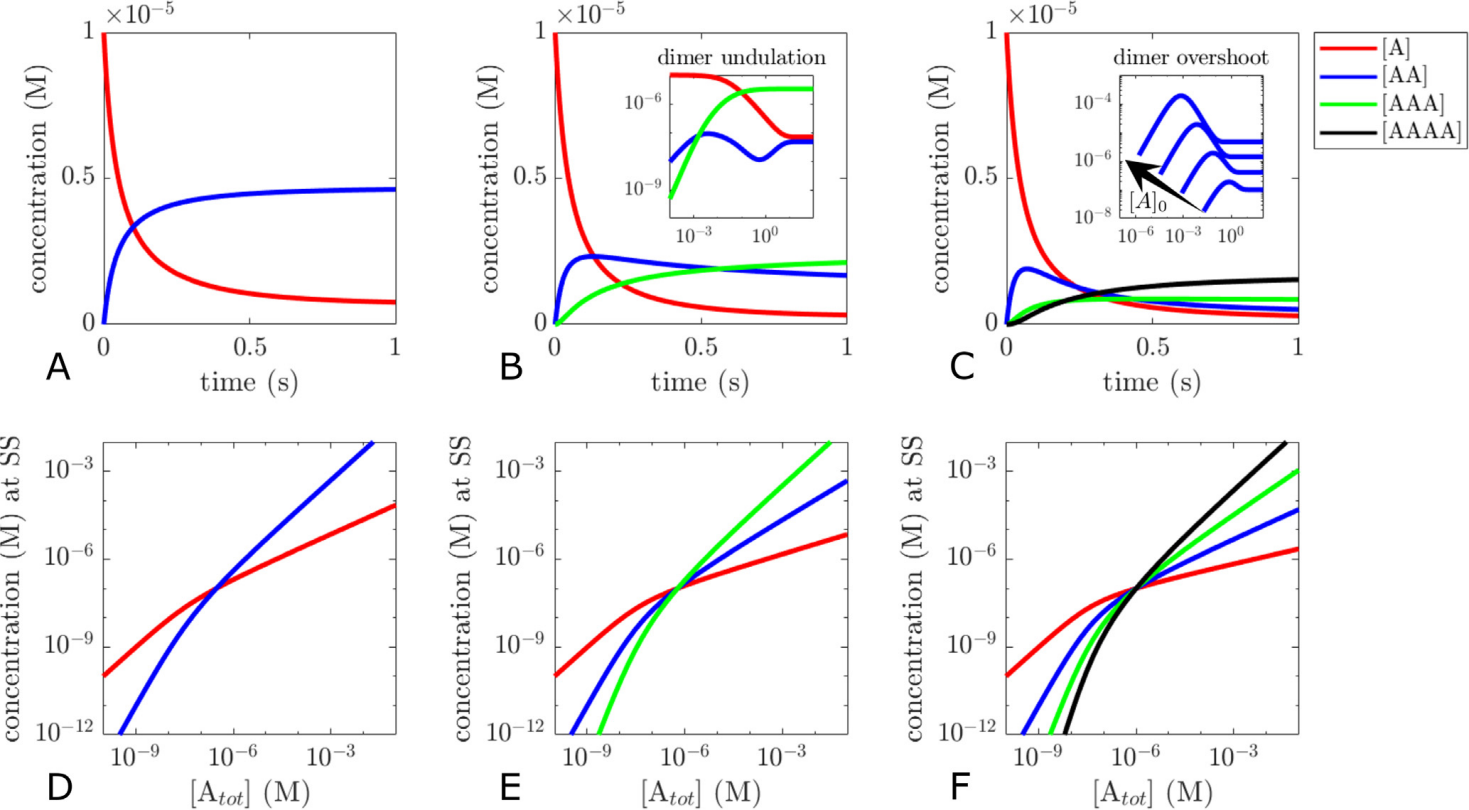
Time course simulations (A–C) and steady state analysis (D–F) for dimers (A, D), trimers (B, E) and tetramers (C, F). Initial conditions for A–C: ${\left[A\right]}_{0}=10\;\mu \text{M},{\left[\mathrm{AA}\right]}_{0}={\left[\mathrm{AAA}\right]}_{0}={\left[\mathrm{AAAA}\right]}_{0}=0\;\text{M}$. Parameters: (A and D) $k_{1}=10^{6}\;{\text{mol}}^{-1}\;{\text{s}}^{-1},k_{2}=0.1\;{\text{s}}^{-1}$ , (B and E) $k_{1}=k_{3}=10^{6}\;{\text{mol}}^{-1}\;{\text{s}}^{-1},k_{2}=k_{4}=0.1\;{\text{s}}^{-1}$ , (C and F) $k_{1}=k_{3}=k_{5}=k_{7}=10^{6}\;{\text{mol}}^{-1}\;{\text{s}}^{-1}$ (C inset: $k_{3}=10^{8}\;{\text{mol}}^{-1}\;{\text{s}}^{-1}$), $k_{2}=k_{4}=k_{6}=k_{8}=0.1\;{\text{s}}^{-1}$.

Time course simulations of the system with initial conditions ${\left[A\right]}_{0}=A_{\mathrm{tot}}=10\;\mu \text{M}$ show association dynamics typical for binary protein–protein interactions in the case of dimerisation, whereas trimerisation and tetramerisation reactions exhibit a transient overshoot of dimers followed by a slower decrease of dimers and an increase in trimers and tetramers, respectively (Fig. 2A–C). Amplitude and position of such overshoots strongly depend on the monomer concentration at the beginning of the reaction (Fig. 2C, inset). More complex dynamics such as dampened oscillations or undulations on different time scales are possible (Fig. 2B, inset). If the individual oligomeric species possess different biological functionality, such dynamics could be a mechanism for dynamic signal encoding as will be outlined in the discussion in more detail.

Numerical steady state analysis shows that the dose–response curves for the individual species meet in a single intersection point (Fig. 2D–F), mirroring the assumption that all reactions have the same $K_{d}$ value. Local sensitivity analysis at $A_{\mathrm{tot}}=10\mathrm{nM}$ with 2% perturbation yields relative sensitivities $\frac{\mathrm{dS}}{{\mathrm{dA}}_{\mathrm{tot}}}\frac{A_{\mathrm{tot}}}{S}$ (where *S* is the steady state concentration of either A,…,AAAA) of 0.87 for monomers and 1.76 for dimers in the dimerisation model, 2.58 for trimers in the trimersation model and 3.46 for tetramers in the tetramerisation model. The analysis confirms again that oligomerisation at concentrations below the $K_{d}$ can lead to modest ultrasensitivity in response to changes in total protein concentration, and that ultrasensitivity can increase with higher number of protomers per complex (as can also be seen from the increasing slopes in Fig. 2E, F) (Buchler and Louis, 2008).

**Fig. 3:**
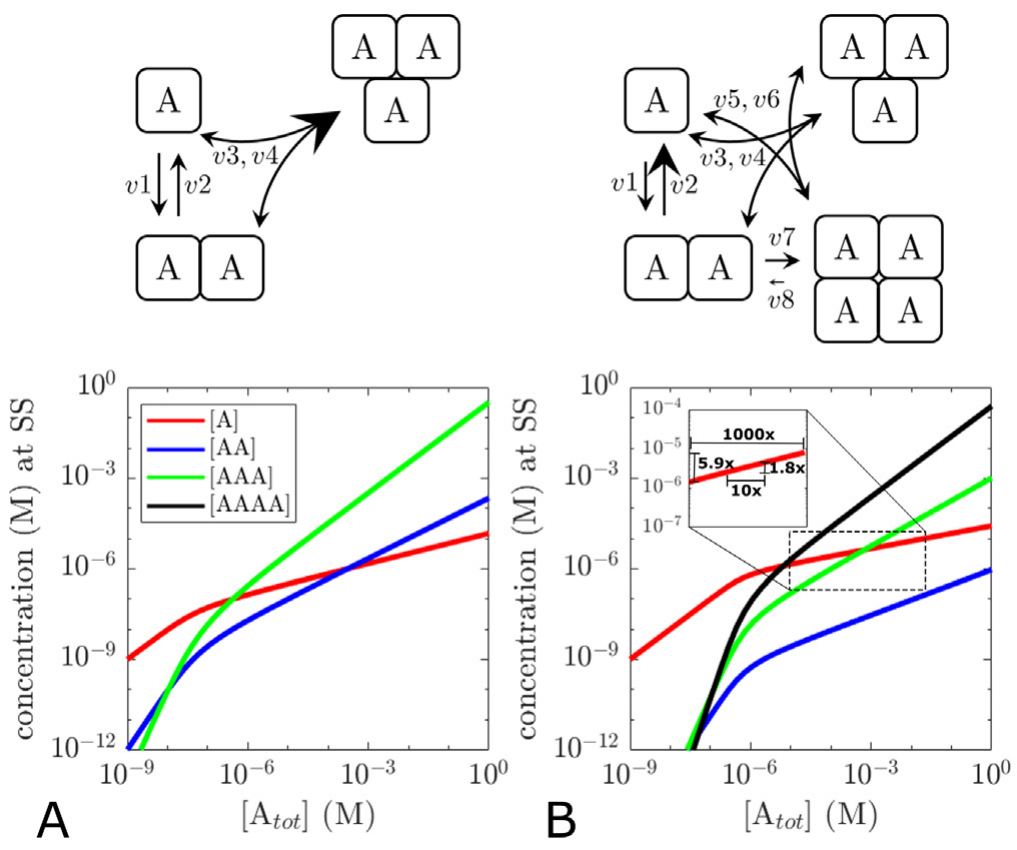
Steady state analysis of trimerisation and tetramerisation models with varied parameters. The relative change of parameters is visualised in the upper reaction schemes. Parameters: (A) $k_{1}=10^{6}\;{\text{mol}}^{-1}\;{\text{s}}^{-1},k_{3}=10^{8}\;{\text{mol}}^{-1}\;{\text{s}}^{-1},k_{2}=k_{4}=0.1\;{\text{s}}^{-1}$, (B) $k_{1}=3.2\times 10^{6}\;{\text{mol}}^{-1}\;{\text{s}}^{-1},k_{2}=2400\;{\text{s}}^{-1}$$k_{3}=3.45\times 10^{6}\;{\text{mol}}^{-1}\;{\text{s}}^{-1},k_{4}=0.083\;{\text{s}}^{-1},k_{5}=4.8\times 10^{6}\;{\text{mol}}^{-1}\;{\text{s}}^{-1},k_{6}=0.525\;{\text{s}}^{-1},k_{7}=3\times 10^{6}\;{\text{mol}}^{-1}\;{\text{s}}^{-1},k_{8}=1.0525\times 10^{-5}\;{\text{s}}^{-1}$.

For many proteins able to form higher order oligomers, the presence of a single or a small subfraction of possible oligomeric species often dominates over other potential intermediate species (Powers and Powers, 2003), indicating that oligomerisation is often cooperative and that $K_{d}$ values differ for the individual reactions. Varying the model parameters in a way to favour formation of the highest order oligomer in the trimerisation and tetramerisation model (e.g. by increasing association rate constants) can reproduce the dominance of the highest order oligomer over large concentration ranges (Fig. 3A, B). This also leads to a shift of intersection points, resulting in different apparent $K_{d}$ values between the individual intermediate oligomerisation reactions. Tweaking of the parameters allows to shift the curves for each individual species into almost any direction (data not shown). Parameter variation also highlights the flipside of oligomeric ultrasensitivity. If we consider the monomer concentration at higher total protein concentrations in the inset of Fig. 3B, it becomes apparent that oligomerisation can be an efficient homeostatic regulatory mechanism of the monomer concentration (relative sensitivity of 0.25 for monomers at $A_{\mathrm{tot}}=100\;\mu \text{M}$). This would be plausible in situations where monomers are the biologically active species. Note that this mechanism does not require a complex feedback organisation typically associated with homeostasis (Cannon, 1929, Tyson et al., 2003).

### 2.2 Considering post-translational modification of homo-oligomers

Just like non-oligomeric proteins, oligomeric proteins are subject to various post-translational modifications such as phosphorylation, ubquitinylation, lipidation and others. Sometimes these modifications can regulate the equilibria between monomeric and oligomeric species via conformational changes or sterical hindrance. Other times these modifications are irrelevant to the protein’s oligomerisation behaviour. Even accounting merely for a single PTM makes model formulation of anything higher than dimers unlikely more difficult due to the combinatorial expansion of potential oligomerisation routes.

Unfortunately, combinatorial expansion is not the only challenge when PTMs of oligomers are considered: it is remarkably easy to slip into thermodynamical inconsistency even with models based purely on conventional mass action kinetics. The reason for this is that PTMs induce a combinatorial asymmetry: for a monomer, a single PTM site results in an either modified or unmodified state. For a n-tamer with the same PTM site, however, n+1 possibilities to modify the n-tamer emerge (assuming that only the total number of PTMs is relevant). A model of dimerisation, for example, thus has to account for a single monomeric and a single dimeric species if no PTMs are considered. If PTMs are considered, however, there are two monomeric species and three dimeric species (unmodified, singly modified, fully modified) to be accounted for. The model thus ‘grows’ asymmetrically on the n-tamer site. A more detailed description of the problem and how to avoid it can be found in Supplementary section 1. In the following, we shall only consider models which have been balanced according to the procedure outline there.

To proceed with model formulation, let us suppose an oligomeric protein can be modified by a PTM at a single site. For the sake of simplicity we assume that the site lies remote from the oligomerisation interface and does not alter any of the reaction parameters. Let $A^{*},{\mathrm{AA}}^{*},{\mathrm{AA}}^{**},\ldots$denote modified monomers, dimers with one and dimers with two modified protomers and so forth. We assume molecules such as $A^{*}A$ and ${\mathrm{AA}}^{*}$ are identical due to symmetry. Let E1 and E2 be a modifying and a demodifying enzyme for *A*’s PTM site, respectively, both of which operate by a non-cooperative, irreversible and distributive mechanism. We assume that all molecular species, regardless the number of their protomers, are (de-)modified with the same kinetic parameters, i.e. the oligomeric state does not influence the (de-)modification reactions. These assumptions reflect a situation in which a PTM does not induce conformational changes and lies remote from the oligomerisation interface, allowing the enzymes to access the PTM site equally in all oligomeric species. We therefore expect the individual monomeric and oligomeric species to compete for enzymes E1 and E2. In situations with multiple competing substrates $S_{1},\ldots ,S_{n}$, an irreversible Michaelis–Menten type rate law of the form:

$$v_{i}=\frac{V_{\max}S_{i}}{K_{m_{i}}\left(1+\underset{j\in J⧹\{i\}}{\sum }\frac{S_{j}}{K_{m_{j}}}\right)+S_{i}},$$

where J={1,…,n}, can be employed to describe the rate of consumption $v_{i}$ of substrate $S_{i}$ (Schäuble et al., 2013). That is, the individual substrates act as competitive inhibitors for each other. Like previous studies, we use a Michaelis–Menten type rate law to limit the number of parameters and reactions to be modelled (Markevich et al., 2004, Conradi and Mincheva, 2014). Note, however, that modelling a specific signalling pathway with low substrate concentrations can require mass action kinetics (Salazar and Höfer, 2009). We are now able to formulate reaction schemes, reaction rates and model equations.

**Fig. 4:**
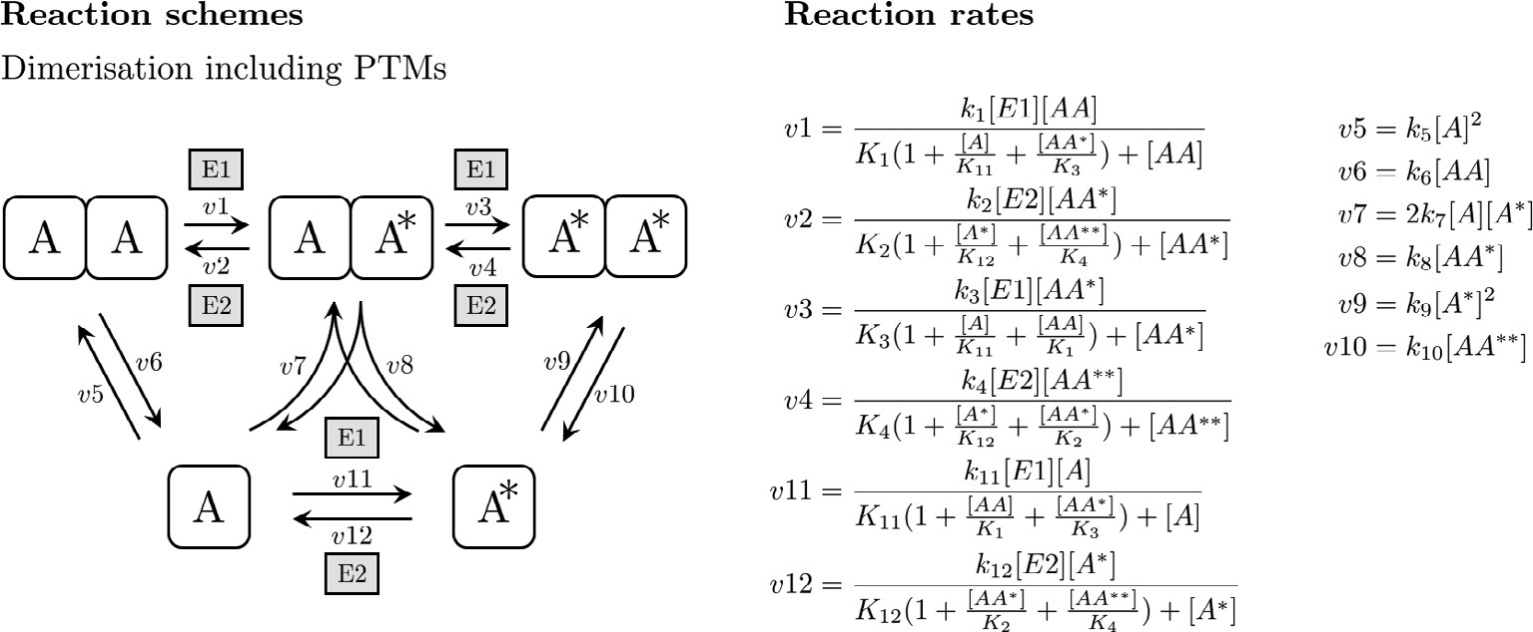
Reaction scheme and reaction rates for the mass action kinetics model of dimerisation including reversible post-translational modification of a single site.

Fig. 4A shows the reaction scheme and rate expressions for a dimerisation model based on mass action kinetics for oligomerisation and mentioned Michaelis–Menten type rate law for addition and removal of PTMs. The equations are:

$$\begin{array}{cc} & \frac{d}{\mathrm{dt}}[A]=2\cdot v6+v8+v12-2\cdot v5-v7-v11\\ & \frac{d}{\mathrm{dt}}[A^{*}]=v8+2\cdot v10+v11-v7-2\cdot v9-v12\\ & \frac{d}{\mathrm{dt}}[\mathrm{AA}]=v2+v5-v1-v6\\ & \frac{d}{\mathrm{dt}}[{\mathrm{AA}}^{*}]=v1+v4+v7-v2-v3-v8\\ & \frac{d}{\mathrm{dt}}[{\mathrm{AA}}^{**}]=v3+v9-v4-v10 \end{array}$$

Rate v7 has been balanced according to the procedure outlined in Supplementary section 1.

### 2.3 Ultrasensitivity and bistability via pseudo-multisite modification

**Fig. 5:**
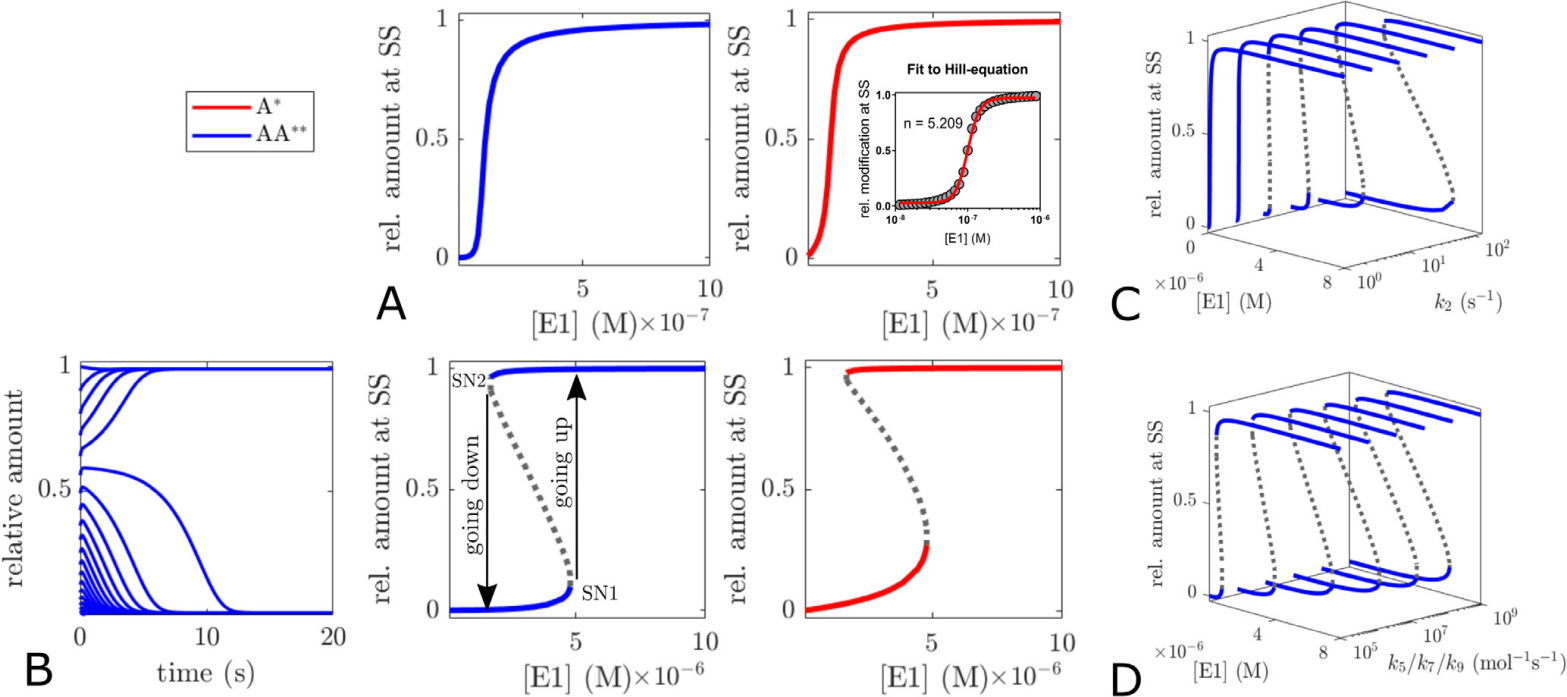
Ultrasensitivity and Bistability of the modification response. Parameters and initial conditions: $k_{5}=k_{7}=k_{9}=10^{7}\;{\text{mol}}^{-1}\;{\text{s}}^{-1},k_{6}=k_{8}=k_{10}=10\;{\text{s}}^{-1},k_{1}=k_{2}=k_{3}=k_{4}=k_{11}=k_{12}=1\;{\text{s}}^{-1},K_{1}=K_{2}=K_{3}=K_{4}=K_{11}=K_{12}=1\;\mu \text{M},A_{\mathrm{tot}}=10\;\mu \text{M},[E2]=0.1\;\mu \text{M}$ (A) fractional modification of both monomers and dimers in response to increasing concentrations of modifying enzyme E1 is notably ultrasensitive. (B) left, time course simulations demonstrate that the approached steady state is determined by the initial conditions if demodification is assumed to be cooperative. Parameters: $k_{2}=100\;{\text{s}}^{-1},[E1]=3\;\mu \text{M}$, different fractional modification at t = 0). (B) middle and right, bifurcation diagrams show identical parameter values for saddle node bifurcations of dimer and monomer modification. Unstable steady states are indicated by dotted lines. (C,D) The bistable range of the modification response increases with stronger kinetic cooperativity (C) and dimer formation (D).

We will begin exploring the steady state behaviour in the presence of (de-)modifying enzymes E1,E2 using the balanced dimer model as an example. The relative fraction of modified dimer and monomer shows pronounced ultrasensitivity in response to increasing concentrations of modifying enzyme E1 (Fig. 5A). On closer examination, this is not very surprising. Apart from some degree of zero-order ultrasensitivity arising from enzyme saturation (Goldbeter and Koshland, 1981), oligomerisation additionally creates a substrate competition situation between monomeric and oligomeric species for (de-)modification and provides pseudo-multisites for PTMs (i.e. multiple protomers with identical PTM sites). Both motifs are capable of generating ultrasensitivity (Salazar and Höfer, 2006, Ferrell et al., 2014).

Moreover, multisite modification can in principle generate bi- or multistability if there is a sufficient asymmetry in the sequential modification cycles, i.e. if either the demodification and/or the modification steps exhibit kinetic cooperativity (Markevich et al., 2004, Ortega et al., 2006, Thomson and Gunawardena, 2009). For dual-site modification of monomeric proteins, Conradi and Mincheva have proven that in general, bistability must occur for some concentrations of demodifying and modifying enzyme if the product of the rate constants for the first modification and demodification steps is smaller than the product of the rate constants for the second modification and demodification steps (Conradi and Mincheva, 2014). Without considering the oligomeric nature, introducing positive kinetic cooperativity for the demodification of the dimer, i.e. assuming $k_{2}>k_{4}$, would fulfill this requirement. Indeed, implementing this assumption leads to bistability with respect to the modification status in the dimer model (Fig. 5B). The range over which bistability occurs depends both on the degree of kinetic cooperativity and on the extend of dimerisation (Fig. 5C, D). As the bistable range increases with the number of cooperative modification steps (Ortega et al., 2006), the likelihood for a bistable PTM status will also increase with higher order oligomers.

Interestingly, not only the dimer, but also the monomer modification exhibits bistability even without multiple sites for PTMs. This becomes less surprising if one considers that the dimer is in equilibrium with the monomer, allowing modified dimers to dissociate into monomers. Furthermore, when dimers are completely (de-)modified, substrate competition for (de-)modification of the monomer abates, allowing for more monomer (de-)modification.

**Fig. 6:**
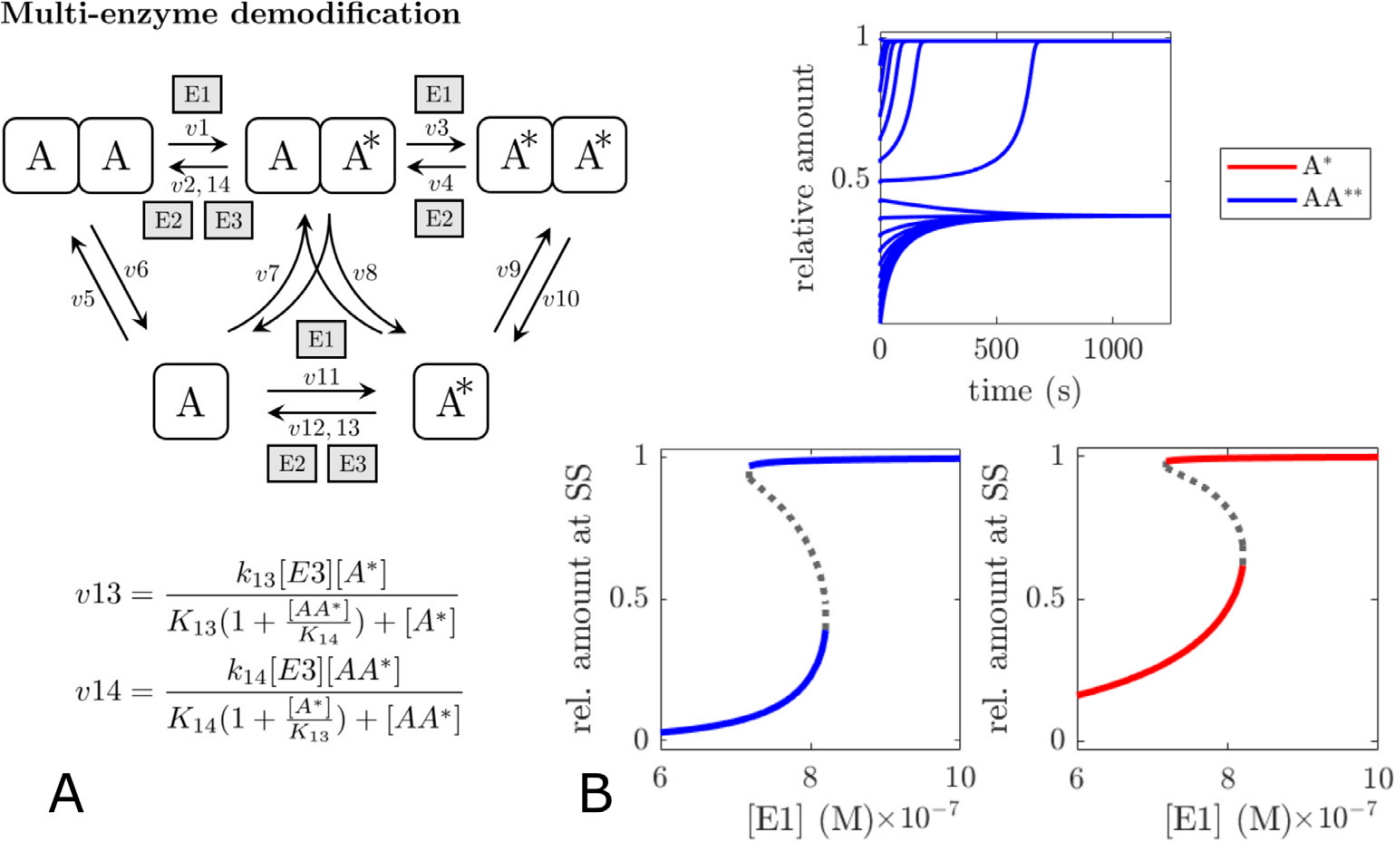
Bistability through multi-enzyme regulation of the modification status of oligomers. (A) scheme of the (balanced) dimer model with additional demodification enzyme E3 which can not catalyse the first step of the dimer demodification. (B) time course simulations and bifurcation plots demonstrating bistability in the dimer model. Parameters: $[E2]=10\mathrm{nM},[E3]=0.1\;\mu \text{M},k_{13}=0.1\;{\text{s}}^{-1},k_{14}=100\;{\text{s}}^{-1},K_{13}=10\;\mu \text{M},K_{14}=1\;\mu \text{M}$, other parameters as specified for Fig. 5.

While perhaps not uncommon, kinetic cooperativity might not be the only way to realise bistability in (pseudo-)multisite PTM systems. From a biochemical point of view, asymmetry in the (de-)modification rate of a multisite PTM protein could effectively be realised, too, if one of the (de-)modification steps would also be catalysed by another enzyme E3. Let us, for instance, assume that in a dually modified dimer each PTM mutually prevents (e.g. due to sterical reasons) access to the other PTM for demodifying enzyme E3. Only when one of the PTMs has already been removed by demodifying enzyme E2 (which we assume to catalyse PTM removal from the singly and dually modified dimer equally well), can E3 bind to the singly modified dimer and catalyse the last demodification step. Assuming that E3 can also catalyse demodification of the modified monomer, the scheme for the updated dimer model is shown in Fig. 6A. Using the updated dimer model it is not difficult to find parameter values that lead to bistability (Fig. 6B), showing that multi-enzyme regulation can be an effective alternative for realising the asymmetry required for bistability in multisite PTM systems.

## 3 Discussion

### 3.1 Complex dynamics and steady state behaviour

Even simple homo-oligomerisation systems can in principle be capable of surprisingly complex behaviour. Dynamical phenomena such as transient overshoots of dimers followed by a slower increase in higher order oligomers will be relevant to proteins which are not in a constitutive monomer/oligomer equilibrium. Examples include membrane receptors which oligomerise upon ligand binding (Klemm et al., 1998) or proteins which oligomerise upon recruitment to a membrane. If dimers and higher order oligomers have different downstream signalling functions, such transients could be an effective way to encode the duration of the input signal (e.g. ligand presence or membrane recruitment) and thereby lead to different cellular responses for short and prolonged stimuli. The tumor suppressor p53 is a relevant example of a protein with different biological activity for different oligomeric species (Rajagopalan et al., 2011). As p53 is also involved in dynamic signal encoding leading to different cell-fate decisions (Sonnen and Aulehla, 2014), it is tempting to speculate that some of this could be the result of oligomerisation. Another promising candidate for dynamic signal encoding through oligomerisation could be the EGF-receptor for which dimers, trimers and tetramers have been described (Furuuchi et al., 2007, Gan et al., 2007). A considerable list of higher-order homo-oligomers for which various intermediate forms have been observed (and thereby might also be candidates for dynamic signal encoding) can be found in Selwood and Jaffe (2012).

In addition to the previously described but modest ultrasensitivity through oligomerisation (Buchler and Louis, 2008, Hsu et al., 2016), we have seen that oligomerisation could also be an effective homeostatic regulatory mechanism to keep monomer concentration in a narrow range. In contrast to the dynamical phenomena, this more likely applies to proteins which are in a constitutive monomer/oligomer equilibrium. Recently, Frieden proposed oligomerisation as metabolic control mechanism (Frieden, 2019). Given that many enzymes oligomerise, monomer-homeostasis could be a good example. If enzyme function is inhibited e.g. by active site obstruction in an oligomeric complex (Matthews and Sunde, 2012), monomer-homeostasis could ensure a nearly constant performance of a metabolic activity over a wide range of total protein concentration (and therefore cellular conditions such as starvation or different cell cycle phases).

So far, few oligomeric proteins have been studied experimentally extensively enough to validate scenarios such as depicted in Fig. 3B. Since individual species concentrations in the homeostatic scenario often differ by ⩾2 orders of magnitude, experimental testing of such behaviour would at least require to determine the equilibrium distribution of monomeric and oligomeric species over several orders of magnitude of total protein concentration. Ideally, this would be complemented by kinetic data on oligomer (dis-)assembly. Both types of experiments can be technically challenging and likely need to be analysed via model fitting (Kanno and Levitus, 2014, Parsons et al., 2019).

### 3.2 Combinatorial complexity

As the order of oligomers increases and/or PTMs are taken into account, the number of species and possible reactions quickly grows. This is a typical situation of ‘combinatorial explosion’ which poses a significant challenge for many signal transduction models (Hlavacek et al., 2003, Stefan et al., 2014). If PTMs are not considered and only one oligomeric species is relevant, oligomerisation pathways can be approximated via *generalised mass action* rate laws (i.e. power-laws) provided that the range of total concentrations is sufficiently restricted (data not shown).

Upon inclusion of PTMs, the combinatorial expansion of possible oligomerisation routes posed another unanticipated challenge: ensuring thermodynamic consistency of the model. The rate balancing procedure described in the Supplementary material offers a solution which is straightforward to apply to mass action kinetics models. An open question is how this procedure fares if PTMs do affect oligomerisation parameters. A plausible conjecture would be that once the balancing coefficients have been introduced into the rate equations, changing parameter values for individual reactions will not affect thermodynamic consistency.

For practical purposes, modelling higher-order oligomers with multiple PTM sites will generally require implicit modelling approaches. Rule-based modelling, for instance, has been applied successfully for modelling EGF-receptor oligomerisation (Kozer et al., 2013).

### 3.3 Bistability

Ultrasensitivity and bistability are important properties of signal transduction networks for cellular decision making, allowing to respond in a switch-like, binary and sometimes irreversible fashion. Oligomerisation can also lead to ultrasensitivity and bistability by providing pseudo-multisite complexes (i.e. complexes with multiple identical PTM sites). Given previous work on ultrasensitivity and bistability arising via multisite modification from the Kholodenko lab and others (Markevich et al., 2004, Ortega et al., 2006, Salazar and Höfer, 2006, Conradi and Mincheva, 2014), this possibility seems obvious from a biochemical point of view, yet, has not been appreciated before. An interesting and unique twist of this motif is that the bistability resulting from modification of the oligomer extends to the monomer due to intrinsic substrate competition and because both species are in equilibrium with each other. We also demonstrated that kinetic cooperativity of multisite modification systems is not a requirement for bistability. If multiple enzymes regulate the modification steps and if some can only catalyse a subset of the individual modification steps, this leads effectively to the same kinetic asymmetry (Ortega et al., 2006, Conradi and Mincheva, 2014) as kinetic cooperativity. While oligomers might be particularly suited for this mechanism due to their symmetrical quaternary structure, bistability through multi-enzyme regulation could in principle arise in any multisite PTM system.

The relevance of these findings is that they significantly expand the range of contexts in which one should look for biochemical ‘switches’ as both homo-oligomerisation and multi-enzyme regulation are extremely common. Phosphatases, for example, are known to often act promiscuously on multiple substrates (Shi, 2009). As a consequence, many phosphorylation sites can be dephosphorylated by multiple phosphatases, creating potential situations in which bistability could occur. Alternatively, multi-enzyme regulation of the modification rather than demodification steps is also conceivable. Phosphorylating a protomer within a dimer, for example, could lead to a new binding site for a second kinase facilitating phosphorylation of the same residue in the other protomer. The combination of both mechanisms, oligomerisation and multi-enzyme regulation, therefore represents an interesting novel signalling motif that does not require feedback or kinetic cooperativity to generate bistable responses.

Other biologically relevant examples in which bistability are predicted to play important roles are small GTPase networks (Barr, 2013, Conte-Zerial et al., 2008), some for which bistability has recently been demonstrated experimentally (Byrne et al., 2016, Bezeljak et al., 2020). Interestingly, many small GTPases homo-dimerise (Chen et al., 2016, Daitoku et al., 2001, Zhang and Zheng, 1998) and are typically inactivated (i.e. converted to the GDP-bound form) by multiple GTPase activating proteins (GAPs) (Müller and Goody, 2018, Lawson and Ridley, 2018). Thus, the motifs presented in this paper might plausibly cause or contribute to the emergence of bistability in small GTPase networks.

### 3.4 Conclusion

We have demonstrated that homo-oligomers, making up approximately 30–50% of the proteome (Lynch, 2012, Marsh and Teichmann, 2015), offer an even greater variety of regulatory mechanisms than previously appreciated. Since two thirds of all enzymes homo-oligomerise (Marianayagam et al., 2004) and since about 44% of homo-oligomers are involved in signal transduction (Fig. S5), these mechanisms could be relevant to many cellular signalling pathways. Furthermore, it may partly explain why homo-oligomerisation is so commonly found throughout evolution. Hopefully, the presented findings will be helpful to modellers interested in homo-oligomeric signalling proteins and stimulate experimental research into signalling processes to which the presented findings might apply. Supplementary section 7 provides an overview of techniques and suggested experimental designs that could be deployed to test presented predictions.

## 4 Methods

Details on the computational procedures can be found in the Supplementary material.

## Competing Interests

The author has no competing interests to declare.

## CRediT authorship contribution statement

**Daniel Koch:** Conceptualization, Investigation, Formal analysis, Writing - original draft.
