# Supplementary material for: Homo-Oligomerisation in Signal Transduction: Dynamics, Homeostasis, Ultrasensitivity, Bistability
Source: J Theor Biol. 2020 Aug 21;499:110305. doi: 10.1016/j.jtbi.2020.110305 (PMC7327509; doi:10.1016/j.jtbi.2020.110305)
Supplement: Supplementary file 1 [file mmc1.pdf]

# - Supplementary Information -

Homo-Oligomerisation in Signal Transduction: Dynamics, Homeostasis, Ultrasensitivity, Bistability

Daniel Koch
orcid.org/0000-0002-4893-1774

Randall Centre for Cell & Molecular Biophysics,
King's College London, London SE1 1UL, United Kingdom
E-mail: daniel.koch@kcl.ac.uk

Contents

1 Ensuring thermodynamical consistency in models of homo-oligomers subject to post-translational modification 2

2 Proof: conservation of oligomerisation rates 10

3 Reaction rates and ODEs for unbalanced trimerisation model including PTMs 13

4 Balancing rates of the trimerisation model including PTMs 15

5 Computational methods 17

6 Other supplementary figures 17

7 Experimental techniques and designs for testing model predictions 19

8 References 23

---

1 Ensuring thermodynamical consistency in models of homo-oligomers subject to post-translational modification

As outlined in the main text, consideration of PTMs in models of homo-oligomerisation can lead to thermodynamic inconsistencies. Before we incorporate PTMs into our models we will therefore formulate some biochemical intuitions and expectations which will later guide us to avoid such inconsistencies. Let us suppose an oligomeric protein can be modified by a PTM at a single site. For the sake of simplicity, we assume that the site lies remote from the oligomerisation interface and does not alter any of the reaction parameters. Intuitively, we would then expect the following to be true:

(a) The association rate constants (a1), dissociation rate constants (a2) and therefore equilibrium and dissociation constants (a3) are identical for the following oligomerisation reactions:

- only unmodified protein
- only modified protein
- modified with unmodified protein

(b) If the modifying enzyme is added to an equilibrated mixture of completely unmodified monomers and oligomers, both monomers and oligomers will be modified over time, yet the total concentrations of monomers and oligomers remains constant.

(c) There are $\binom{2+n-1}{n}$ possibilities to combine unmodified/modified monomers into symmetric n-tamers. Thus, if equal parts of unmodified/modified monomers are mixed to form n-tamers, the isoform distribution of n-tamers with $0 \leq i \leq n$ modified subunits will be binomial.

The notation and assumptions introduced in the main text are repeated for convenience: Let $A^*, AA^*, AA^{**}, ...$ denote modified monomers, dimers with one and dimers with two modified protomers and so forth. Due to assumed symmetry, molecules such as A^*A and AA^* are identical. Keeping the assumption that each oligomeric species is formed through binary association reactions, it follows from (a1)-(c1) that each pair of molecular species X,Y which is able to associate in the absence of any PTMs is also able to associate with identical reaction parameters regardless of how many protomers of X or Y are modified. We will assume that there is a modifying enzyme $E1$ and a demodifying enzyme $E2$ which operate by a non-cooperative, irreversible and distributive mechanism and that all molecular species, regardless the number of their protomers, are (de-)modified with the same kinetic parameters, i.e. the oligomeric state does not influence the (de-)modification reactions. These assumptions reflect

the situation where a PTM does not induce conformational changes and lies remote from the oligomerisation interface, allowing the enzymes to access the PTM site equally in all oligomeric species. We therefore expect the individual monomeric and oligomeric species to compete for enzymes $E1$ and $E2$. In situations with multiple competing substrates $S_1, ..., S_n$ an irreversible Michaelis-Menten type rate law of the form:

$$v_i = \frac{V_{max} S_i}{K_{m_i}(1 + \sum_{j \in J \setminus \{i\}} \frac{S_j}{K_{m_j}}) + S_i},$$

where $J = \{1, ..., n\}$, can be employed to describe the rate of consumption v_i of substrate S_i [1]. That is, the individual substrates act as competitive inhibitors for each other. We are now able to formulate reaction schemes, reaction rates and model equations.

Figure S1A shows the reaction scheme and rate expressions for the dimerisation model based on mass action kinetics for oligomerisation and mentioned Michaelis-Menten type rate law for addition and removal of PTMs. The equations are:

$$\frac{d}{dt}[A] = 2 \cdot v6 + v8 + v12 - 2 \cdot v5 - v7 - v11, \quad \frac{d}{dt}[A^*] = v8 + 2 \cdot v10 + v11 - v7 - 2 \cdot v9 - v12,$$

$$\frac{d}{dt}[AA] = v2 + v5 - v1 - v6, \quad \frac{d}{dt}[AA^*] = v1 + v4 + v7 - v2 - v3 - v8,$$

$$\frac{d}{dt}[AA^{**}] = v3 + v9 - v4 - v10.$$

Figure S1B shows the reaction scheme for the trimerisation model including PTMs. See supplementary section 3 for reaction rates and model equations. Note that the reaction scheme for trimerisation and a single PTM is already more complex than the tetramerisation scheme without PTMs. For oligomerisation, we will use the following parameter values: $10^7 mol^{-1}s^{-1}$ for dimer formation, $10^9 mol^{-1}s^{-1}$ for trimer formation, $10s^{-1}$ for dissociation of dimers and trimers. We chose catalytic rate constants to be $k_{cat} = 1s^{-1}$ and Michaelis constants to be $K_m = 1\mu M$ for all (de-)modification reactions in both models.

Let us first consider the effects of adding catalytic concentrations of E1 to an equilibrated mixture of monomers and oligomers. Interestingly, as the modification reaction proceeds, transient changes in the total concentrations of monomeric and oligomeric species occur (Figure S2A; the total concentrations being the sum of concentrations of all modified isoforms for a given monomeric or oligomeric species). The amplitude and direction of these transients appear to be influenced by the maximum number of protomers. Intuitively, the phenomenon results from the accessibility of additional oligomerisation routes during the modification reaction. Consider for example the dimerisation reaction scheme (Figure S1A). If A is either completely unmodified or modified, a single reaction route between a monomeric and a dimeric species is available. If a mixture of A and A^* is present, a third route appears: the reaction between A and A^* to

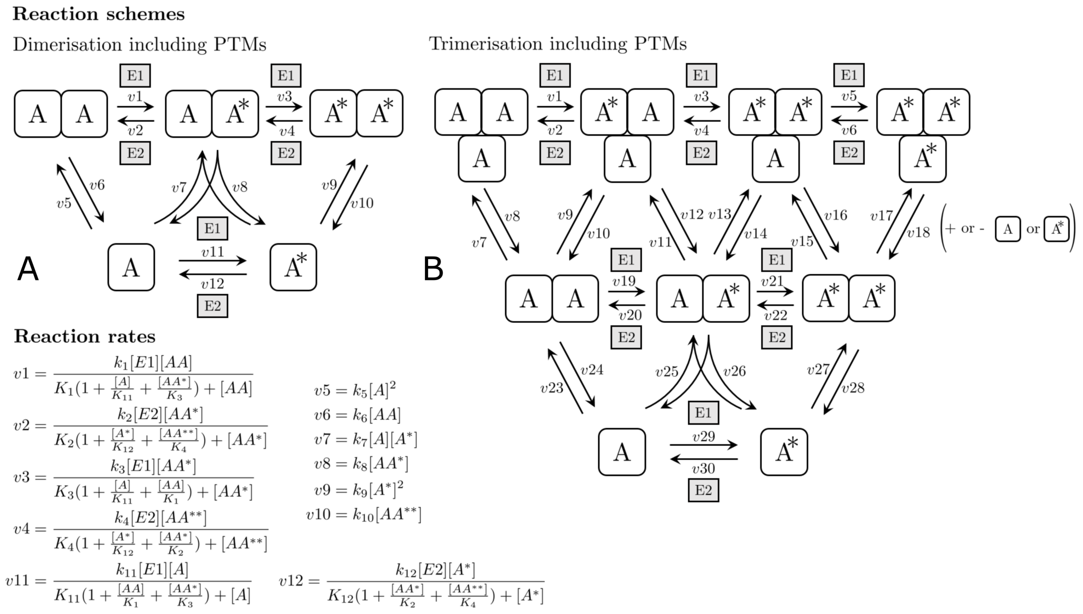

Figure S1
Reaction schemes for the mass action kinetics models of dimerisation (A) and trimerisation (B) including reversible post-translational modifications. See supplementary section 3 for reaction rates and ODEs of the trimersation model.

AA^*. We would therefore expect that decreasing the velocity of oligomerisation/dissociation or increasing the speed of modification would diminish the effect, as both changes would limit A's capacity to change its oligomerisation state during the transition time from completely unmodified protein to complete modification. Indeed, while decreasing the velocity of oligomerisation/dissociation reduces the amplitude, increasing the enzyme concentration limits the peak width of the amplitude (Figure S4).

Clearly, these transient changes contradict expectation (b) and no experimental data so far (as far as the author is aware) reported transient alterations of the oligomerisation state upon post-translation without affecting association/dissociation rates. To further explore the models and to test whether the transient changes could be a modelling artefact, let us see what happens if equimolar mixtures of modified and unmodified A are simulated to steady state. According to (a3) we would expect K_d values to be identical to that of completely unmodified and completely modified A. We would furthermore expect a binomial distribution of modified oligomers according to (c). However, both models show altered K_d values (Figure S2B) and a uniform distribution of modified oligomers (Figure S2C). Taken together, this suggests that the transient changes are indeed a modelling artefact resulting from thermodynamic inconsistency.

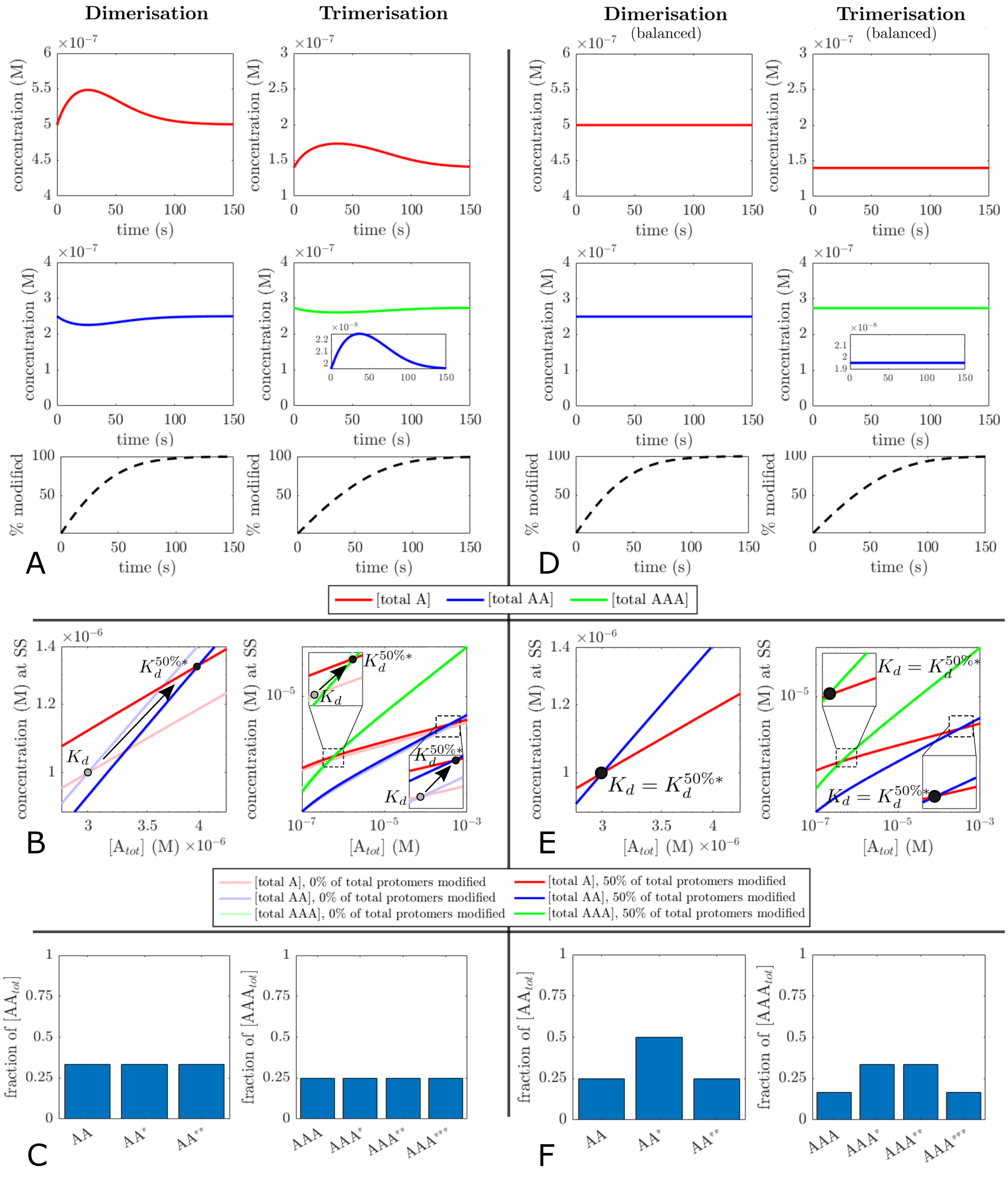

Figure S2
Behaviour of di- and trimerisation models upon post-translational modification. (A,D) timecourses of total monomer and total oligomer concentrations after addition of $50nM$ $[E1]$ starting with $A_{tot} = 10\mu M$ at equilibrium. Dimerisation rate constants: $10^7 mol^{-1}s^{-1}$, trimerisation rate constants: $10^9 mol^{-1}s^{-1}$, dissociation rate constants of dimers and trimers: $10s^{-1}$. Black-dotted curves show progress of the modification reaction. (B,E) apparent dissociation constants and (C,F) distribution of PTM isoforms of the highest order oligomer in equimolar mixtures of modified and unmodified protein at equilibrium.

Where did we go astray if using simple mass action kinetics, arguably the most established deterministic modelling approach for chemical reactions, leads to thermodynamic inconsistency? The answer is: naively deriving ordinary differential equations by applying mass action kinetics and stoichiometrical balancing to reaction schemes such as in Figure S1. To circumvent this issue, we need to introduce a more general notation for oligomeric species and formulate a further expectation.

Let A_n^m denote a m-times modified n-tamer, i.e. an oligomeric complex with $n \in \mathbb{N}^{\geq 1}$ identical subunits of which $0 \leq m \leq n$ carry a PTM at site x. Let $j \in \mathbb{N}^{\geq 1}$ be the number of reversible bimolecular association reactions between lower-order complexes A_r^p and A_s^q able to form A_n^m, where $r + s = n$.

Next, let $I(A_n) = \{\{r_1, s_1\}, \{r_2, s_2\}, ...\}$ define the set of actually occurring combinations of oligomeric orders of A_n's educts $A_{r_i}^p$ and $A_{s_i}^q$, where $p + q \leq n$ and $r_i + s_i = n$. For example, if a hexamer can be assembled from dimers with tetramers and from monomers with pentamers (of any PTM status), $I(A_6)$ would be the set $\{\{2,4\},\{1,5\}\}$.

From (a1) and (a2) it follows that all forward steps between $A_{r_i}^p$ and $A_{s_i}^q$ producing A_n^m have the same association rate constant k_i and each corresponding reverse step has the same dissociation rate constant k_i' for all possible values of m, p and q. Unaltered rate constants imply unaltered equilibrium constants. Therefore, we expect that

(d) the PTM status is irrelevant for the equilibrium. Thus, the equilibrium is solely determined by the total concentrations of each n-tamer, i.e. the sum of all PTM-isoforms of A_n:

$$[A_{n,tot}] = \sum_{m=0}^{n} [A_n^m]$$

In other words: from the perspective of the oligomerisation equilibrium, all PTM isoforms of a n-tamer are treated as a single species. Figure S3 illustrates these definitions and the equilibrium situation.

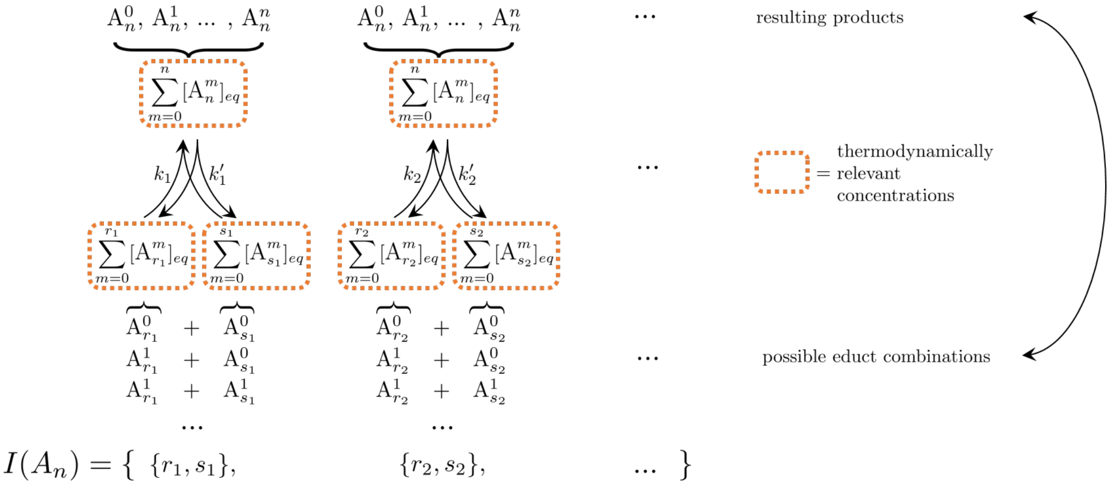

Figure S3
Illustration of the n-tamer equilibrium situation if a PTM does not influence the oligomerisation reaction. According to expectation (d) in the main text, we expect the equilibrium to be solely determined by the total n-tamer concentrations $\sum_{m=0}^{n}[A_n^m]_{eq}$ if the PTM status does not influence oligomerisation. The set $I(A_n)$ denotes the actually occurring combinations of oligomeric orders of A_n's educts, i.e. $|I(A_n)|$ is the number of reversible reactions that would produce/consume A_n if PTMs were not taken into consideration.

We can now formulate the following principle:

Conservation of oligomerisation rates

If a PTM does not influence the parameters of an oligomerisation reaction, the sum of all rates v_i, $1 \le i \le j$, of reactions leading to a n-tamer A_n of any modification status is equal to the association rates based on the total concentrations of A_n's educts (i.e. all modification isoforms). Conversely, the sum of all rates v_i', $1 \le i \le j$, of reactions dissociating A_n of any modification status is equal to the dissociation rate based on A_n's total concentration. That is at all times

$$\sum_{i=1}^{j} v_i = \sum_{i\in I}(k_i \cdot \sum_{m=0}^{r}[A_{r_i}^m] \cdot \sum_{m=0}^{s}[A_{s_i}^m]),$$

and

$$\sum_{i=1}^{j} v_i' = \sum_{i\in I}(k_i' \cdot \sum_{m=0}^{n}[A_n^m]).$$

We call the right-hand side of each identitiy the *effective rates.*

The principle's name is chosen in analogy to the conservation of mass as it conserves the reaction rates at given total concentrations of educts regardless the distribution of PTM isoforms. Although it might appear complicated, it is straightforward to illustrate the principle using

dimerisation as an example. Applying it to Figure S1A yields $I(A_2) = \{\{1,1\}\}$ and thus

$$v5 + v7 + v9 = k_1([A] + [A^*])^2 \tag{1}$$

and

$$v6 + v8 + v10 = k_1'([AA] + [AA^*] + [AA^{**}]). \tag{2}$$

How does this principle help us to avoid thermodynamic inconsistency? The principle relates the j reaction rates from the reaction scheme (left-hand side (LHS) of (1) and (2)) to the thermodynamically expected effective rates (right-hand side (RHS) of (1) and (2)). Instead of assigning *a priori* rate expressions based on mass action kinetics, we will assign rates only after making sure the principle is not violated. In the first step of this check, we will expand the RHS of the rate conservation identity. In a second step, we will substitute the rates on the LHS with their mass action kinetics expression. Next, we will compare both sites: if they are equal, all rates can readily be identified with their mass action kinetics expression. If they are not equal, we balance the terms on the LHS where the discrepancy occurs by introducing coefficients that ensure the validity of the identity. Lastly, we identify the respective rates with the balanced terms. Let us illustrate this using equations (1) and (2) derived from the dimerisation scheme:

(1) → *expanding RHS, substituting LHS:*

$$\begin{aligned} &k_1[A]^2 + k_1[A][A^*] + k_1[A^*]^2 \\ &= k_1[A]^2 + 2k_1[A][A^*] + k_1[A^*]^2 \end{aligned}$$

→ *balancing deviating terms in LHS:*

$$\begin{aligned} &k_1[A]^2 + \underline{\mathbf{2}} \cdot k_1[A][A^*] + k_1[A^*]^2 \\ &= k_1[A]^2 + 2k_1[A][A^*] + k_1[A^*]^2 \end{aligned}$$

→ *assigning reaction rates:*

$$\begin{aligned} &v5 = k_1[A]^2, v7 = 2k_1[A][A^*] \\ &v9 = k_1[A^*]^2 \end{aligned}$$

(2) → *expanding RHS, substituting LHS:*

$$\begin{aligned} &k_1'[AA] + k_1'[AA^*] + k_1'[AA^{**}] \\ &= k_1'[AA] + k_1'[AA^*] + k_1'[AA^{**}] \end{aligned}$$

→ *no balancing necessary*

→ *assigning reaction rates:*

$$v6 = k_1'[AA], v8 = k_1'[AA^*]$$
$$v10 = k_1'[AA^{**}]$$

It is straightforward to apply the same procedure to the trimerisation model (cf. supplementary section 4). Other things being equal, the dimerisation and trimerisation model updated with the balanced rate expressions exhibit neither transient changes in the oligomerisation upon modification (Figure S2D), nor shifts in the apparent K_d of equimolar mixtures of unmodified/modified A (Figure S2E). The distribution of PTM isoforms at equilibrium is binomial (Figure S2F). Taken together, this indicates that the balanced models are indeed thermodynamically consistent.

The postulated rate conservation principle is no fundamental law and can readily be proven using the principle of detailed balance [5] as lemma (cf. suppl. section 2).

For a more intuitive understanding of this balancing procedure, it might be helpful to appreciate its similarity to stoichiometric balancing. Consider, for example, the chemical equation for the reaction of oxygen with hydrogen to water:

$$O_2 + H_2 \rightarrow H_2O$$

As the reader will have spotted, there are two oxygen atoms on the LHS, but only one on the RHS of the equation. As this violates the law of conservation of mass, we need to balance the equation by adding stoichiometric coefficients:

$$O_2 + \underline{\mathbf{2}}H_2 \rightarrow \underline{\mathbf{2}}H_2O$$

The rate balancing procedure presented in this paper is conceptually identical: As the PTM is assumed not to influence oligomerisation, we know that total formation and dissociation rates of oligomers are conserved; they must be the same as for unmodified protein. However, the PTM increases the number of possible oligomeric species due to combinatorial expansion. This expansion is asymmetric because there are more possibilities to combine modified and unmodified subunits to a n-tamer the higher its order: two for a monomer, three for a dimer, four for a trimer and so forth (cf. Figure S1B). This creates additional oligomerisation routes and alters the net rates of oligomer formation and dissociation, thereby violating the rate conservation principle. To avoid this, we need to balance this purely combinatorial effect by introducing balancing coefficients for the rate expressions.

2 Proof: conservation of oligomerisation rates

To show the validity of the principle of conservation of oligomerisation rates, we require the principle of detailed balance. While it has often been treated as a fundamental postulate, it can be derived from microscopic reversibility in physics, which is why we treat it as a lemma [3,4].

Lemma: principle of detailed balance

> "When a system is at equilibrium this means that the forward rate of a molecular process has to be equal to the reverse rate of that process. Applying this to a chemical reaction observed on a macroscopic scale means that at equilibrium, the forward rate of each step is equal to the reverse rate of that step; this is the principle of detailed balance." [5]

For a system in which some molecular species S can be formed by $j \in \mathbb{N}^{\geq 1}$ reversible reactions this means at equilibrium:

$$\sum_{i=1}^{j} v_i = \sum_{i=1}^{j} v_i', \tag{3}$$

where v_i is the forward rate and v_i' the reverse rate of reaction i.

Although the multitude of sums might give the impression of being complicated, the idea for the principle's proof itself is simple: we find that applying the principle of detailed balance and the assumption that the PTM does not influence oligomerisation (expectation (e) from the main text) to the n-tamer equilibrium concentrations results in the same equations as given by the postulated conservation principle but restricted to equilibrium. We can then prove the principle by contradiction. If we assume that the conservation principle would be false and apply the resulting inequalities to the equilibrium situation, we obtain direct contradictions to the before derived equations describing the equilibrium. For convenience, the principle to be proven is repeated in summarized form below:

Conservation of oligomerisation rates

Let A_n be an oligomeric complex with $n \in \mathbb{N}^{\geq 1}$ protomers[1] which can be formed through $j \in \mathbb{N}^{\geq 1}$ reversible bimolecular association reactions between lower-order complexes $A^p_{r_i}$ and $A^q_{s_i}$, where $p + q \leq n$ and $r_i + s_i = n$, $i \in I(A_n)$. The PTM does not influence the oligomerisation reaction.

It follows that at all times, the sum of all rates v_i, $1 \leq i \leq j$, of reactions leading to A_n of any modification status is the association rate based on the total concentrations (i.e. all modification isoforms) of A_n's educts. The sum of all rates v'_i, $1 \leq i \leq j$, of reactions dissociating A_n of any modification status is equal to the dissociation rate based on A_n's total concentration. That is

$$\sum_{i=1}^{j} v_i = \sum_{i \in I} (k_i \cdot \sum_{m=0}^{r_i} [A^m_{r_i}] \cdot \sum_{m=0}^{s_i} [A^m_{s_i}]), \tag{4}$$

and

$$\sum_{i=1}^{j} v'_i = \sum_{i \in I} (k'_i \cdot \sum_{m=0}^{n} [A^m_n]). \tag{5}$$

Proof: For the construction of the contradiction it is necessary to consider the situation at equilibrium. By assumption, the PTM does not influence oligomerisation. From (a1) and (a2) it follows that all forward steps between $A^p_{r_i}$ and $A^q_{s_i}$ producing A^m_n have the same association rate constant k_i and each corresponding reverse step has the same dissociation rate constant k'_i for all possible values of m, p and q. According to (d), equilibrium is only determined by the total concentration of each oligomeric complex, i.e. the relevant educt concentrations are $\sum_{m=0}^{r_i} [A^m_{r_i}]_{eq}$ and $\sum_{m=0}^{s_i} [A^m_{s_i}]_{eq}$, whereas the relevant product concentration is $\sum_{m=0}^{n} [A^m_n]_{eq}$. Thus, the expressions for forward and reverse rates are

$$k_i \cdot \sum_{m=0}^{r_i} [A^m_{r_i}]_{eq} \cdot \sum_{m=0}^{s_i} [A^m_{s_i}]_{eq}$$

and

$$k'_i \cdot \sum_{m=0}^{n} [A^m_n]_{eq},$$

respectively, for all $i \in I$. Applying the principle of detailed balance (3) to the formation and dissociation of A_n at equilibrium and substituting above rate expressions therefore gives:

$$\sum_{i=1}^{j} v_i = \sum_{i \in I} (k_i \cdot \sum_{m=0}^{r} [A^m_{r_i}]_{eq} \cdot \sum_{m=0}^{s} [A^m_{s_i}]_{eq}) = \sum_{i \in I} (k'_i \cdot \sum_{m=0}^{n} [A^m_n]_{eq}) = \sum_{i=1}^{j} v'_i. \tag{6}$$

[1] Monomers are considered as oligomers for purely formal reasons.

Separating (4) into its first and last identity yields

$$\sum_{i=1}^{j} v_i = \sum_{i \in I} (k_i \cdot \sum_{m=0}^{r} [A_{r_i}^m]_{eq} \cdot \sum_{m=0}^{s} [A_{s_i}^m]_{eq}), \tag{7}$$

and

$$\sum_{i=1}^{j} v_i' = \sum_{i \in I} (k_i' \cdot \sum_{m=0}^{n} [A_n^m]_{eq}). \tag{8}$$

Let us for a moment assume that the conservation of oligomerisation rates would not be true. We have to distinguish three cases: equation (4) is false, equation (5) is false, both (4) and (5) are false.

Case 1: equation (4) is false, i.e.

$$\sum_{i=1}^{j} v_i \neq \sum_{i \in I} (k_i \cdot \sum_{m=0}^{r} [A_{r_i}^m] \cdot \sum_{m=0}^{s} [A_{s_i}^m]),$$

$$\text{at equilibrium} \quad \sum_{i=1}^{j} v_i \neq \sum_{i \in I} (k_i \cdot \sum_{m=0}^{r} [A_{r_i}^m]_{eq} \cdot \sum_{m=0}^{s} [A_{s_i}^m]_{eq}),$$

in contradiction to (7).

Case 2: equation (5) is false, i.e.

$$\sum_{i=1}^{j} v_i' \neq \sum_{i \in I} (k_i' \cdot \sum_{m=0}^{n} [A_n^m]),$$

$$\text{at equilibrium} \quad \sum_{i=1}^{j} v_i' \neq \sum_{i \in I} (k_i' \cdot \sum_{m=0}^{n} [A_n^m]_{eq}),$$

in contradiction to (8).

Case 3: Both (4) and (5) are false. The contradiction follows from case 1 and case 2.

□

3 Reaction rates and ODEs for unbalanced trimerisation model including PTMs

This section contains the reaction rates and ODEs for the trimer reaction scheme presented in Figure S1B.

Reaction rates

association & dissociation reactions

$$v7 = k_7[A][AA] \qquad v8 = k_8[AAA]$$
$$v9 = k_9[A^*][AA] \qquad v10 = k_{10}[AAA^*]$$
$$v11 = k_{11}[A][AA^*] \qquad v12 = k_{12}[AAA^*]$$
$$v13 = k_{13}[A^*][AA^*] \qquad v14 = k_{14}[AAA^{**}]$$
$$v15 = k_{15}[A][AA^{**}] \qquad v16 = k_{16}[AAA^{**}]$$
$$v17 = k_{17}[A^*][AA^{**}] \qquad v18 = k_{18}[AAA^{***}]$$
$$v23 = k_{23}[A]^2 \qquad v24 = k_{24}[AA]$$
$$v25 = k_{25}[A][A^*] \qquad v26 = k_{26}[AA^*]$$
$$v27 = k_{27}[A^*]^2 \qquad v28 = k_{28}[AA^{**}]$$

modification reactions

$$v1 = \frac{k_1[E1][AAA]}{K_1(1 + \frac{[A]}{K_{29}} + \frac{[AA]}{K_{19}} + \frac{[AA^*]}{K_{21}} + \frac{[AAA^*]}{K_3} + \frac{[AAA^{**}]}{K_5}) + [AAA]}$$
$$v3 = \frac{k_3[E1][AAA^*]}{K_3(1 + \frac{[A]}{K_{29}} + \frac{[AA]}{K_{19}} + \frac{[AA^*]}{K_{21}} + \frac{[AAA]}{K_1} + \frac{[AAA^{**}]}{K_5}) + [AAA^*]}$$
$$v5 = \frac{k_5[E1][AAA^{**}]}{K_5(1 + \frac{[A]}{K_{29}} + \frac{[AA]}{K_{19}} + \frac{[AA^*]}{K_{21}} + \frac{[AAA]}{K_1} + \frac{[AAA^*]}{K_3}) + [AAA^{**}]}$$
$$v19 = \frac{k_{19}[E1][AA]}{K_{19}(1 + \frac{[A]}{K_{29}} + \frac{[AA^*]}{K_{21}} + \frac{[AAA]}{K_1} + \frac{[AAA^*]}{K_3} + \frac{[AAA^{**}]}{K_5}) + [AA]}$$
$$v21 = \frac{k_{21}[E1][AA^*]}{K_{21}(1 + \frac{[A]}{K_{29}} + \frac{[AA]}{K_{19}} + \frac{[AAA]}{K_1} + \frac{[AAA^*]}{K_3} + \frac{[AAA^{**}]}{K_5}) + [AA^*]}$$
$$v29 = \frac{k_{29}[E1][A]}{K_{29}(1 + \frac{[AA]}{K_{19}} + \frac{[AA^*]}{K_{21}} + \frac{[AAA]}{K_1} + \frac{[AAA^*]}{K_3} + \frac{[AAA^{**}]}{K_5}) + [A]}$$

demodification reactions

$$v2 = \frac{k_2[E2][AAA^*]}{K_2(1 + \frac{[A^*]}{K_{30}} + \frac{[AA^*]}{K_{20}} + \frac{[AA^{**}]}{K_{22}} + \frac{[AAA^{**}]}{K_4} + \frac{[AAA^{***}]}{K_6}) + [AAA^*]}$$
$$v4 = \frac{k_4[E2][AAA^{**}]}{K_4(1 + \frac{[A^*]}{K_{30}} + \frac{[AA^*]}{K_{20}} + \frac{[AA^{**}]}{K_{22}} + \frac{[AAA^*]}{K_2} + \frac{[AAA^{***}]}{K_6}) + [AAA^{**}]}$$
$$v6 = \frac{k_6[E2][AAA^{***}]}{K_6(1 + \frac{[A^*]}{K_{30}} + \frac{[AA^*]}{K_{20}} + \frac{[AA^{**}]}{K_{22}} + \frac{[AAA^*]}{K_2} + \frac{[AAA^{**}]}{K_4}) + [AAA^{***}]}$$
$$v20 = \frac{k_{20}[E2][AA^*]}{K_{20}(1 + \frac{[A^*]}{K_{30}} + \frac{[AA^{**}]}{K_{22}} + \frac{[AAA^*]}{K_2} + \frac{[AAA^{**}]}{K_4} + \frac{[AAA^{***}]}{K_6}) + [AA^*]}$$
$$v22 = \frac{k_{22}[E2][AA^{**}]}{K_{22}(1 + \frac{[A^*]}{K_{30}} + \frac{[AA^*]}{K_{20}} + \frac{[AAA^*]}{K_2} + \frac{[AAA^{**}]}{K_4} + \frac{[AAA^{***}]}{K_6}) + [AA^{**}]}$$
$$v30 = \frac{k_{30}[E2][A^*]}{K_{30}(1 + \frac{[AA^*]}{K_{20}} + \frac{[AA^{**}]}{K_{22}} + \frac{[AAA^*]}{K_2} + \frac{[AAA^{**}]}{K_4} + \frac{[AAA^{***}]}{K_6}) + [A^*]}$$

The ODE system is:

$$\begin{aligned}
\frac{d}{dt}[A] &= v8 + v12 + v16 + 2 \cdot v24 + v26 + v30 - v7 - v11 - v15 - 2 \cdot v23 - v25 - v29 \\
\frac{d}{dt}[A^*] &= v10 + v14 + v18 + 2 \cdot v28 + v26 + v29 - v9 - v13 - v17 - 2 \cdot v27 - v25 - v30 \\
\frac{d}{dt}[AA] &= v8 + v10 + v20 + v23 - v7 - v9 - v19 - v24 \\
\frac{d}{dt}[AA^*] &= v12 + v14 + v19 + v22 + v25 - v11 - v13 - v20 - v21 - v26 \\
\frac{d}{dt}[AA^{**}] &= v16 + v18 + v21 + v27 - v15 - v17 - v22 - v28 \\
\frac{d}{dt}[AAA] &= v2 + v7 - v1 - v8 \\
\frac{d}{dt}[AAA^*] &= v1 + v4 + v9 + v11 - v2 - v3 - v10 - v12 \\
\frac{d}{dt}[AAA^{**}] &= v3 + v6 + v13 + v15 - v4 - v5 - v14 - v16 \\
\frac{d}{dt}[AAA^{***}] &= v5 + v17 - v6 - v18
\end{aligned}$$

4 Balancing rates of the trimerisation model including PTMs

As for the dimer model, we work under the assumption that all association steps leading to an oligomeric complex A_n have the same rate constant k_i and all dissociation steps have the same rate constant k_i'. As trimers can only be formed by association of dimers and monomers (i.e. $I(A_3) = \{\{1,2\}\}$), we leave out the index $i \in I$ for convenience. We begin the rate balancing of the trimerisation model by noticing that the lower part of the trimerisation scheme depicted in Figure S1B is structurally identical to the dimerisation scheme in Figure S1A. We thus can use the same rate balancing coefficients for the dimerisation steps in the trimer model, i.e. we balance $v25$ by multiplying it with 2 to obtain $v25 = 2k[A][A^*]$. We next apply the rate conservation principle to the formation of trimers and obtain:

$$v7 + v9 + v11 + v13 + v15 + v17 = k[A_t][AA_t] = k([A] + [A^*]) \cdot ([AA] + [AA^*] + [AA^{**}])$$

→ *expanding RHS, substituting LHS:*

$$\begin{aligned} &k[AA][A] + k[AA][A^*] + k[AA^*][A] + k[AA^*][A^*] + k[AA^{**}][A] + k[AA^{**}][A^*] \\ &= k[AA][A] + k[AA][A^*] + k[AA^*][A] + k[AA^*][A^*] + k[AA^{**}][A] + k[AA^{**}][A^*] \end{aligned}$$

→ *no balancing necessary*

→ *assigning reaction rates:*

$$\begin{aligned} v7 &= k[AA][A] \\ v9 &= k[AA][A^*] \\ v11 &= k[AA^*][A] \\ v13 &= k[AA^*][A^*] \\ v15 &= k[AA^{**}][A] \\ v17 &= k[AA^{**}][A^*] \end{aligned}$$

Applying the rate conservation principle to the dissociation of trimers we obtain:

$$v8 + v10 + v12 + v14 + v16 + v18 = k'[AAA_t] = k'([AAA] + [AAA^*] + [AAA^{**}] + [AAA^{***}])$$

→ *expanding RHS, substituting LHS:*

$$\begin{aligned} &k'[AAA] + k'[AAA^*] + k'[AAA^*] + k'[AAA^{**}] + k'[AAA^{**}] + k'[AAA^{***}] \\ &= k'[AAA] + k'[AAA^*] + k'[AAA^{**}] + k'[AAA^{***}] \end{aligned}$$

We notice that the dissociation rates for singly and dually modified trimers appear twice in the LHS, but only once in the RHS. We therefore need to

→ *balance deviating terms in LHS:*

$$k'[AAA] + \mathbf{\frac{1}{2}}k'[AAA^*] + \mathbf{\frac{1}{2}}k'[AAA^*] + \mathbf{\frac{1}{2}}k'[AAA^{**}] + \mathbf{\frac{1}{2}}k'[AAA^{**}] + k'[AAA^{***}]$$
$$= k'[AAA] + k'[AAA^*] + k'[AAA^{**}] + k'[AAA^{***}]$$

→ *assigning reaction rates:*

$$v8 = k'[AAA]$$
$$v10 = \frac{1}{2}k'[AAA^*]$$
$$v12 = \frac{1}{2}k'[AAA^*]$$
$$v14 = \frac{1}{2}k'[AAA^{**}]$$
$$v16 = \frac{1}{2}k'[AAA^{**}]$$
$$v18 = k'[AAA^{***}]$$

5 Computational methods

Presented models have been implemented as MATLAB® scripts for numerical simulation and model analysis. All simulations were performed using the ode23s integrator. Sensitivity analysis has been performed as described in [2]. All model code is accessible in the BioModels database under: https://www.ebi.ac.uk/biomodels/MODEL2003160001.

Bifurcation diagrams have been generated using a custom algorithm that iteratively identifies the unstable steady states. If the algorithm does not converge within the specified number of iterations, it approximates the unstable steady state. The approximation is based on the distribution of concentrations from all time courses taking advantage of the fact that simulations very close to the unstable steady state take a long time until they eventually tip to either of the stable steady states. For more details, please refer to: https://www.ebi.ac.uk/biomodels/MODEL1910220002.

6 Other supplementary figures

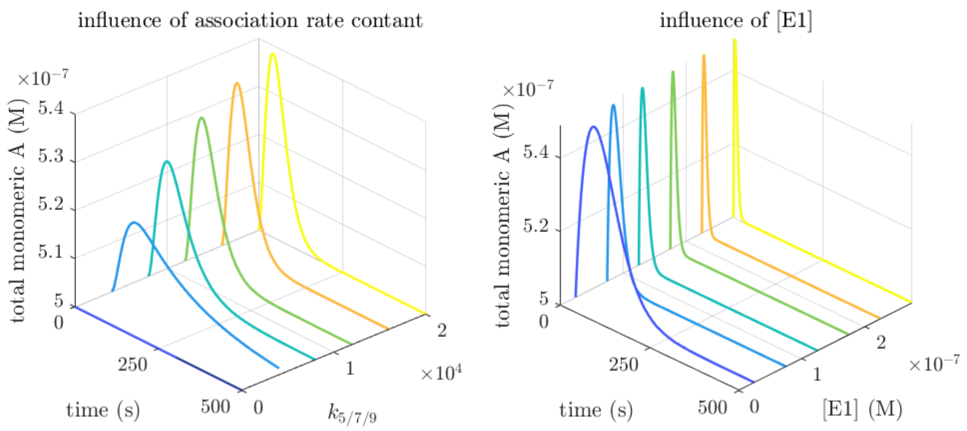

Figure S4
Dependence of transient changes in the mass action dimerisation model on association rate constants and concentration of the modifying enzyme.

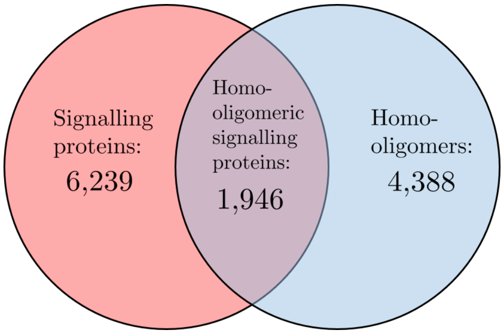

Figure S5
Bioinformatic analysis of the intersection between homo-oligomerisation and signalling. Proteins annotated with the Gene Ontology (GO) term 'Signal Transduction' (GO:0007165) are considered signalling proteins. For proteins which homo-oligomerise, this is estimated from a large human protein-protein interaction network compiled from various databases and published experiments (see [6]). 'self loops' (i.e. edges in the network which connects a protein to itself) are considered as homo-oligomerisation. The number of proteins which homo-oligomerise is simply the number of 'self loops' in this network. The number of oligomeric signalling proteins is then the number of unique protein IDs associated with the above mentioned GO-term that also feature a self loop.

7 Experimental techniques and designs for testing model predictions

Since I am not aware of published experimental data that confirms the presented model predictions for a specific signalling protein or pathway, this section contains a brief overview of suitable methods and experimental designs that could be used to test the model predictions presented in this paper for a specific context.

Methods for detecting the oligomeric state of proteins

A variety of methods for detecting the oligomeric state of proteins exists, ranging from measuring populations of molecules *in vitro* to single molecule techniques allowing to measure individual binding/unbinding events in living cells. For a more detailed discussion of advantages and disadvantages of individual techniques the reader is referred to [7, 8]. An instructive example of a study analysing a protein's oligomeric state using multiple approaches can be found in [9]. The following table gives an overview and refers to reviews or studies which have applied the respective method:

Method	Description	References
Size exclusion chromatography	Simple and widely available method for separating proteins by their hydrodynamic radius. Molecular weight of fractions can be estimated from retention standards, relative oligomer distribution can be approximated by peak integration. When combined with light scattering measurements, absolute molecular weights and thus oligomeric order can be determined. Low temporal resolution, unsuited for complex analytes.	[10, 11]
Analytical ultracentrifugation	Allows for the determination of the absolute molecular weight of oligomeric complexes as well as equilibrium constants of the oligomerisation reaction.	[12]
Native PAGE	Polyacrylamide gel electrophoresis method that preserves the oligomeric state (since samples are not diluted and boiled in SDS sample buffer). If combined with Western-blotting, the oligomeric state of a protein can be determined even in complex solutions such as cell homogenates.	[13, 14]

Method	Description	References
Fluorescence correlation spectroscopy	Powerful technique which allows to determine both rate constants and equilibrium constants of oligomerisation reactions. Applicable both *in vitro* and in live cells. Data analysis can be difficult and might require itself some modelling.	[15–17]
Others	Other techniques which can give information about oligomeric complexes are mass-spectrometry, single-particle tracking and quantitative imaging approaches such as FRET-, BRET- and TIRF-microscopy. More details can be found in the listed references.	[18–21]

Methods for detecting the PTM status of proteins

The probably most common approach for detecting the PTM status of a protein is the use of PTM specific antibodies which are commercially available for many better characterised proteins. If used for Western-blotting in combination with native PAGE, this should allow to resolve the modification status for different oligomeric species (and even to estimate the fraction of modified protomers if a linear standard is used alongside). In case of phosphorylations, the PTM status can alternatively be determined by using the Phos-tag^{TM} reagent in combination with native PAGE if no PTM-specific antibody is available [22]. If recombinant proteins are used, the phosphorylation status of a protein can also be visualised e.g. by ProQ^{TM} diamond stain.

Analysing the modification status in homogenates can blur the real behaviour of a signalling network taking place at the single-cell level, making it harder to detect phenomena such as ultrasensitivity and bistability [23]. If an antibody is suited for immunofluorescence imaging or FACS, it is possible to resolve the PTM at a single cell level, although the analysis of many conditions (e.g. stimulus doses) can be laborious and material-consuming. This and the limited temporal resolution of immunofluorescence imaging or FACS make these approaches unsuitable for studying the dynamics of a PTM status in live cells. Increasingly sophisticated biosensors for PTMs can overcome this issue and have been applied successfully for quantitative biology purposes [24, 25], although a suitable biosensor design for a protein of interest is a significant challenge on its own.

Experimental designs

This subsection discusses possible experimental designs to detect oligomerisation transients as a means of dynamic signal encoding, monomer homeostasis and ultrasensitivity/bistability in a context of interest.

Oligomerisation transients

As this phenomenon is mainly expected for proteins which oligomerise upon some stimulus (e.g. ligand binding to a receptor or recruitment to a membrane), suitable experiments to test this prediction will begin to monitor the oligomeric state of the protein of interest in the absence of any stimulus. If the protein is approximately at a steady state, the oligomerisation stimulus is added (e.g. a ligand, drug, or liposomes) and changes in oligomeric states are monitored (cf. Figure S6). The phenomenon would be confirmed if at least one oligomeric species shows a significant increase, followed by a significant decrease and increase in higher order oligomers. Key requirements for these experiments are a technique that can distinguish between different oligomeric species with sufficient temporal resolution (fluorescence correlation spectroscopy, single molecule techniques, FRET, perhaps even native PAGE if the process is not too fast) and the possibility to apply the oligomerisation stimulus in the experimental setting.

To evaluate whether the transient is involved in dynamic signal encoding, further functional studies that can assign distinct biological activities to different oligomeric forms (e.g. binding to interaction partners, enzymatic activity) will be necessary.

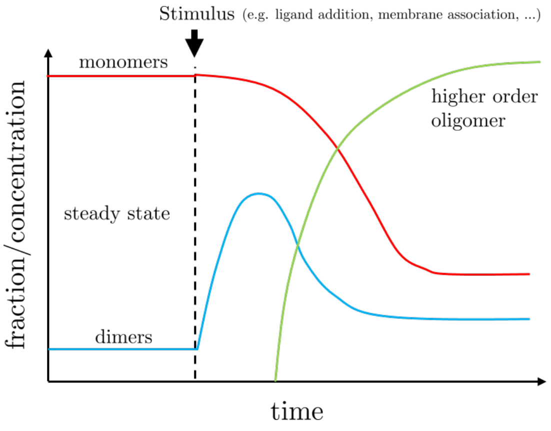

Figure S6
Experimental design to test oligomerisation transients.

Monomer homeostasis

The experimental design for testing whether or not monomer homeostasis occurs is in principle straightforward: measuring the equilibrium concentrations of different oligomeric species at different total protein concentrations. Ideally, this would be done with recombinant purified protein of a known concentration. Size exclusion chromatography, analytical ultracentrifugation, fluorescence correlation spectroscopy and native PAGE should in principle all be suitable techniques for this. Ideally, the range of total protein concentration would be varied by 3-5 orders of magnitude to get a clear picture of the oligomerisation curves on a double-logarithmic

scale. This could pose a challenge for the dynamic range of many methods. If native PAGE is applied as illustrated in Figure S7, the gel can be stained with Oriole^{TM} Fluorescent Gel Stain which can show good linearity for >2 orders of magnitude of total protein amount (personal experience of the author).

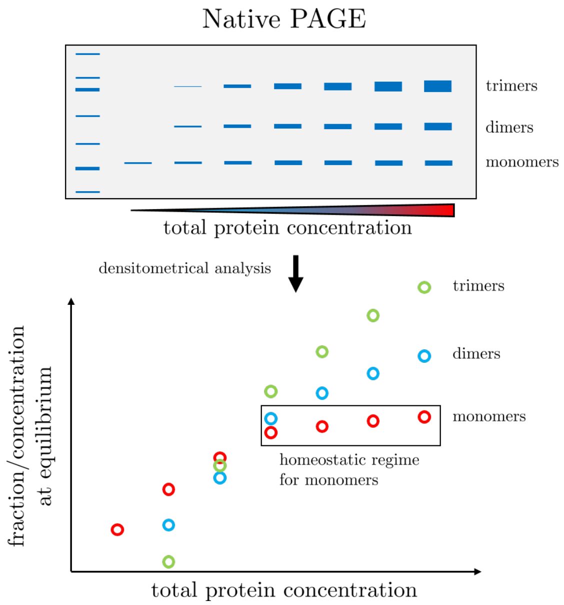

Figure S7
Experimental design to test monomer homeostasis as illustrated for native PAGE.

Ultrasensitivity and bistability

Detecting ultrasensitivity in the PTM status of an oligomer can be simply performed by dose-response experiments in which changes of the stimulus dose lead to changes in the PTM status. The confirmation of bistability ultimately relies on the detection of hysteresis, i.e. dependence of the steady state on the initial conditions of the system. While the general design of such experiments is illustrated in Figure S8, the reader is referred to studies featuring experimental confirmations of bistability for more details [24, 26–28]. Since bistability also occurs for monomers in the models (via equilibration and substrate competition), it may not be necessary to resolve the individual oligomeric species in these experiments, i.e. it would be sufficient to focus on the total modification across oligomeric species.

Ideally, oligomerisation deficient mutants would serve as a negative control. Since ultrasensitivity and bistability in the presented models rely on pseudo-multisite modification in oligomers, preventing oligomerisation should diminish/abolish ultrasensitivity and bistability in the experiments.

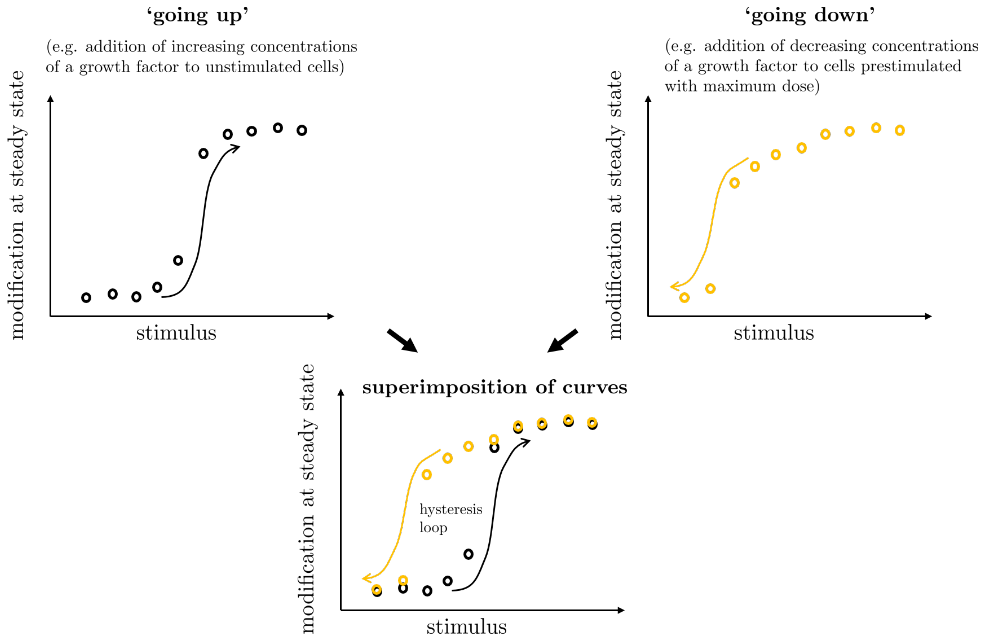

Figure S8
General experimental design to test for bistability: the signalling network has a biochemical 'memory' for its previous modification state (hysteresis).

8 References

1. Schäuble S, Stavrum AK, Puntervoll P, Schuster S, Heiland I. Effect of substrate competition in kinetic models of metabolic networks. FEBS Letters. 2013, 587: 2818–2824.

2. Ingalls B. Mathematical Modeling in Systems Biology: An Introduction. MIT Press, Cambridge MA. 2013.

3. Mahan BH. Microscopic reversibility and detailed balance. An analysis. J Chem Educ. 1975, 52: 299–302.

4. Gorban AN. Detailed balance in micro- and macrokinetics and micro-distinguishability of macro-processes. Results in Physics. 2014, 4: 142–147

5. Alberty RA. Principle of Detailed Balance in Kinetics. J Chem Educ. 2004, 81: 1206–1209.

6. Chung SS, Laddach A, NShaun BT, Fraternali F. Short loop motif profiling of protein interaction networks in acute myeloid leukaemia. bioArxiv. 2018, doi: https://doi.org/10.1101/306886.

7. Gell DA, Grant RP, Mackay JP. The detection and quantitation of protein oligomerization. In: Matthews JM, editor. Protein Dimerization and Oligomerization in Biology. Springer, New York. 2012.

8. Okamoto K, Hiroshima M, Sako Y. Single-molecule fluorescence-based analysis of protein conformation, interaction, and oligomerization in cellular systems. Biophys Rev. 2018, 10: 317–326.

9. Cardarelli S, Miele AE, Zamparelli C, Biagioni S, Naro F, Malatesta F, et al. The oligomeric assembly of the phosphodiesterase-5 is a mixture of dimers and tetramers: A putative role in the regulation of function. BBA - Gen Sub. 2018, 1862: 2183–2190.

10. Moullintraffort L, Bruneaux M, Nazabal A, Allegro D, Giudice E, Zal F, et al. Biochemical and Biophysical Characterization of the Mg^{2+}-induced 90-kDa Heat Shock Protein Oligomers. J Biol Chem. 2010, 285: 15100–15110.

11. Folta-Stogniew E. Oligomeric States of Proteins Determined by Size-Exclusion Chromatography Coupled With Light Scattering, Absorbance, and Refractive Index Detectors. In: Nedelkov D, Nelson RW, editors. New and Emerging Proteomic Techniques. Totowa, NJ: Humana Press; 2006.

12. Taylor IA, Eccleston JF, Rittinger K. Sedimentation Equilibrium Studies. In: Fu H, editor. Protein-Protein Interactions: Methods and Applications. Totowa, NJ: Humana Press; 2004.

13. Wittig I, Braun H-P, Schägger H. Blue native PAGE. Nat Protoc. 2006, 1: 418–428.

14. Niepmann M. Discontinuous native protein gel electrophoresis: pros and cons. Expert Rev Proteomic. 2007, 4: 355–361.

15. Thompson NL, Lieto AM, Allen NW. Recent advances in fluorescence correlation spectroscopy. Curr Opin Struct Biol. 2002, 12: 634–641.

16. Rajagopalan S, Huang F, Fersht AR. Single-Molecule characterization of oligomerization kinetics and equilibria of the tumor suppressor p53. Nucleic Acids Res. 2011, 39:2294–2303.

17. Kanno DM, Levitus M. Protein Oligomerization Equilibria and Kinetics Investigated by Fluorescence Correlation Spectroscopy: A Mathematical Treatment. J Phys Chem B. 2014, 118: 12404–12415.

18. Guo H, An S, Ward R, Yang Y, Liu Y, Guo X-X, et al. Methods used to study the oligomeric structure of G-protein-coupled receptors. Biosci Rep. 2017, 37.

19. Castell OK, Dijkman PM, Wiseman DN, Goddard AD. Single molecule fluorescence for membrane proteins. Methods. 2018, 147: 221–228.

20. Chakraborty H, Chattopadhyay A. Excitements and Challenges in GPCR Oligomerization: Molecular Insight from FRET. ACS Chem Neurosci. 2015, 6: 199–206.

21. Cai X, Bai B, Zhang R, Wang C, Chen J. Apelin receptor homodimer-oligomers revealed by single-molecule imaging and novel G protein-dependent signaling. Sci Rep. 2017, 7: 40335.

22. Deswal S, Beck-García K, Blumenthal B, Dopfer EP, Schamel WWA Detection of phosphorylated T and B cell antigen receptor species by Phos-tag SDS- and Blue Native-PAGE. Immunol Lett. 2010, 130, 51–56.

23. Purvis JE, Lahav G. Encoding and Decoding Cellular Information through Signaling Dynamics. Cell. 2013, 152: 945–956.

24. Mochida S, Rata S, Hino H, Nagai T, Novák B. Two Bistable Switches Govern M Phase Entry. Curr Biol. 2016, 26: 3361–3367.

25. Damayanti NP, Buno K, Narayanan N, Harbin SLV, Deng M, Irudayaraj JMK. Monitoring focal adhesion kinase phosphorylation dynamics in live cells. Analyst. 2017, 142: 2713–2716.

26. Pomerening JR, Sontag ED, Ferrell Jr JE. Building a cell cycle oscillator: hysteresis and bistability in the activation of Cdc2. Nat Cell Biol. 2003, 5: 346–351.

27. Byrne KM, Monsefi N, Dawson JC, Degasperi A, Bukowski-Wills J-C, Volinsky N, et al. Bistability in the Rac1, PAK, and RhoA Signaling Network Drives Actin Cytoskeleton Dynamics and Cell Motility Switches. Cell Syst. 2016, 2: 38–48.

28. Rata S, Rodriguez MFSP, Joseph S, Peter N, Iturra FE, Yang F, et al. Two Interlinked Bistable Switches Govern Mitotic Control in Mammalian Cells. newblock Curr Biol. 2018, 28: 3824-3832.
